# Digital Facilitation to Support Patient Access to Web-Based Primary Care Services: Scoping Literature Review

**DOI:** 10.2196/33911

**Published:** 2022-07-14

**Authors:** Brandi Leach, Sarah Parkinson, Evangelos Gkousis, Gary Abel, Helen Atherton, John Campbell, Christopher Clark, Emma Cockcroft, Christine Marriott, Emma Pitchforth, Jon Sussex

**Affiliations:** 1 RAND Europe Cambridge United Kingdom; 2 University of Exeter Medical School Exeter United Kingdom; 3 University of Warwick Coventry United Kingdom; 4 National Institute of Health and Care Research Collaboration South West Peninsula Patient Engagement Group University of Exeter Medical School Exeter United Kingdom

**Keywords:** web-based health services, primary care, digital facilitation

## Abstract

**Background:**

The use of web-based services within primary care (PC) in the National Health Service in England is increasing, with medically underserved populations being less likely to engage with web-based services than other patient groups. *Digital facilitation*—referring to a range of processes, procedures, and personnel that seek to support patients in the uptake and use of web-based services—may be a way of addressing these challenges. However, the models and impact of digital facilitation currently in use are unclear.

**Objective:**

This study aimed to identify, characterize, and differentiate between different approaches to digital facilitation in PC; establish what is known about the effectiveness of different approaches; and understand the enablers of digital facilitation.

**Methods:**

Adopting scoping review methodology, we searched academic databases (PubMed, EMBASE, CINAHL, Web of Science, and Cochrane Library) and gray literature published between 2015 and 2020. We conducted snowball searches of reference lists of included articles and articles identified during screening as relevant to digital facilitation, but which did not meet the inclusion criteria because of article type restrictions. Titles and abstracts were independently screened by 2 reviewers. Data from eligible studies were analyzed using a narrative synthesis approach.

**Results:**

A total of 85 publications were included. Most (71/85, 84%) were concerned with digital facilitation approaches targeted at patients (promotion of services, training patients to improve their technical skills, or other guidance and support). Further identified approaches targeted PC staff to help patients (eg, improving staff knowledge of web-based services and enhancing their technical or communication skills). Qualitative evidence suggests that some digital facilitation may be effective in promoting the uptake and use of web-based services by patients (eg, recommendation of web-based services by practice staff and coaching). We found little evidence that providing patients with initial assistance in registering for or accessing web-based services leads to increased long-term use. Few studies have addressed the effects of digital facilitation on health care inequalities. Those that addressed this suggested that providing technical training for patients could be effective, at least in part, in reducing inequalities, although not entirely. Factors affecting the success of digital facilitation include perceptions of the usefulness of the web-based service, trust in the service, patients’ trust in providers, the capacity of PC staff, guidelines or regulations supporting facilitation efforts, and staff buy-in and motivation.

**Conclusions:**

Digital facilitation has the potential to increase the uptake and use of web-based services by PC patients. Understanding the approaches that are most effective and cost-effective, for whom, and under what circumstances requires further research, including rigorous evaluations of longer-term impacts. As efforts continue to increase the use of web-based services in PC in England and elsewhere, we offer an early typology to inform conceptual development and evaluations.

**Trial Registration:**

PROSPERO International Prospective Register of Systematic Reviews CRD42020189019; https://www.crd.york.ac.uk/prospero/display_record.php?RecordID=189019

## Introduction

### Background

The use of web-based services within primary care in the National Health Service (NHS) in England is increasing, with 33% of patients registered to use at least one web-based service in January 2021 compared with only 19% in April 2017 [1]. Although still at levels below those found in other countries such as the United States [2], the use of web-based services is likely to grow, given that it is supported by contractual mandates from NHS England [3]; it is part of wider efforts to establish digitally enabled care [4]; and because of increased pressure on health care services, technological progress, and changing public expectations [5]. The use of web-based services has accelerated across primary care in many countries during the COVID-19 pandemic as a means of enabling distanced care [6-10]. Within the NHS, services provided by all primary care practices include booking a consultation (via a practice website or through a web platform linked to a practice website), ordering repeat prescriptions, and accessing electronic health records. Additional services include secure messaging, provision of test results, having a consultation (ie, receiving a response from the practice via SMS text messages, web-based messages, phone calls, or video calls), facilitating access to external resources (eg, referring patients to websites or apps that can augment their care), and providing access to practice websites for informational purposes.

The increased use of web-based services has been shown to benefit patients, general medical practitioners (primary care physicians, known as *general practitioners [GPs]* in the United Kingdom), and other primary care staff through improved communication between patients and GP practices, expanded health and health care knowledge for patients, and improved access to services [11-13]. For GP practices and patients to gain the potential benefits that technological innovation can bring to primary care, patients must be able to, as well as wish to, access and use web-based services [14]. There is emerging evidence that the trend toward web-based interactions creates or exacerbates pre-existing inequalities in access to health care information and services for some patient groups who may not be able, or may not choose, to use or access web-based services [15,16].

A way of supporting the use of web-based services and countering the potential for increasing inequalities may be through *digital facilitation*, which we have defined as “that range of processes, procedures, and personnel which seeks to support NHS patients in their uptake and use of online services” [17]. Digital facilitation ranges from the promotion of web-based services on a practice website to active coaching in the use of web-based services and provision of training and education to practice staff in the use of services so that they can better assist patients [18]. For the purposes of this research, we have not extended the scope of *digital facilitation* to include the facilitation of access to digitally based therapeutic interventions.

Medically underserved and vulnerable populations are less likely than other patient groups to engage in web-based services [2,19]. The reasons for lower engagement in web-based services among medically underserved populations are complex. They include factors focusing on limited access to services (eg, poor internet connection), as well as those affecting motivations to engage (eg, lack of familiarity with the internet, lower health or computer literacy, and lack of trust in web-based information sources) [19-22]. It has been suggested that a way of reducing inequalities related to the use of web-based services in health care may be to actively support vulnerable population groups in accessing and using web-based services through digital facilitation [23]. The digital competence of health care professionals and their acceptance of web-based service provision are also important for the successful implementation of web-based patient services.

### Objectives

Recognizing the lack of understanding of digital facilitation and its role in supporting the use of web-based services in primary care, we conducted a systematic scoping review. We aimed to identify, characterize, and differentiate between different approaches to digital facilitation in primary care; create a typology of these approaches; establish what is known about the effectiveness, perceived advantages, and challenges of different approaches to digital facilitation; examine how they affect inequalities of access to web-based services; and explore factors enabling digital facilitation. We also sought early indications of the extent to which the COVID-19 pandemic might be associated with changing approaches to digital facilitation.

## Methods

### Overview

**Figure 1:**
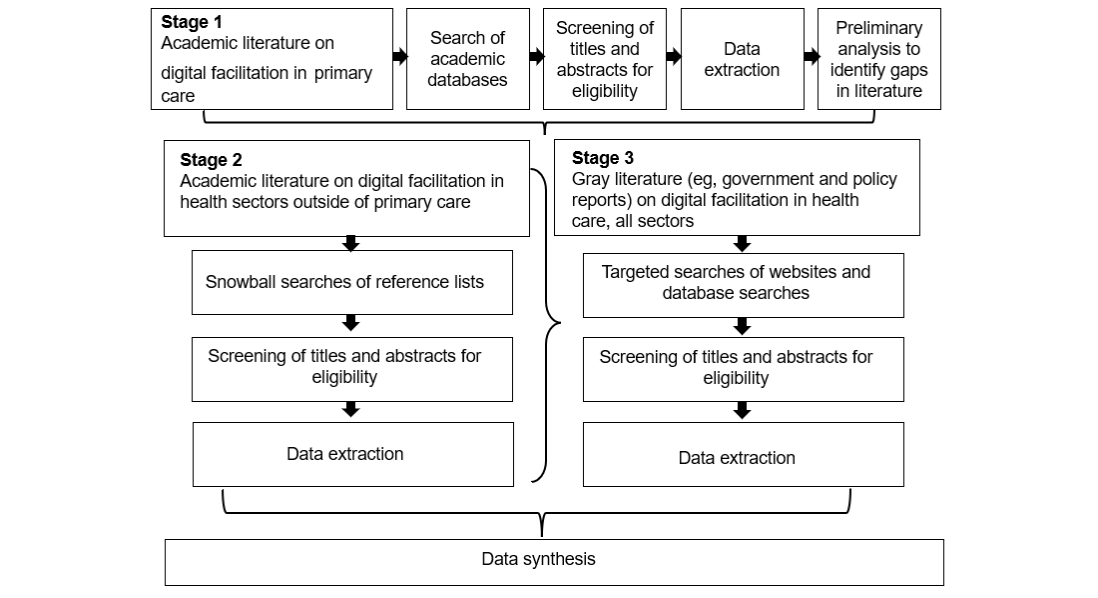
Flow of the literature review process.

We conducted a systematic scoping review of the literature. Scoping reviews are appropriate for clarifying conceptual boundaries on topics, such as digital facilitation, where a concept is new and poorly defined in the literature [24]. The scoping review was conducted in stages (Figure 1) to allow learning from earlier stages to be fed into later stages. The protocol for the study was registered with PROSPERO (International Prospective Register of Systematic Reviews; registration number CRD42020189019) [25].

Our focus is on digital facilitation within primary care in England; however, we also consider digital facilitation in other geographical areas and other health care sectors where there is clear relevance to primary care. Primary care is distinct from other types of health care in that it is typically the patient’s first point of contact within the health system, and the primary care staff is tasked with caring for the patient as a whole rather than focusing on specific conditions [26]. Primary care is also at the center of the NHS’s Digital First plans [27] and faces particular challenges around rising demands in the face of workforce pressures [28]. Although this study focuses on primary care, some findings will also be relevant to wider health care contexts.

### Patient and Public Involvement

This review was conducted in collaboration with a study-specific patient advisory group. The group included patients and caregivers. A total of 2 web-based meetings took place over the course of the project; between meetings, group members were involved via email or on a one-on-one basis. The patient advisory group contributed to the development of the search strategy; operationalization of key terms; discussion of findings, including identified themes and gaps; data synthesis; and report drafting.

### Searches

#### Stage 1: Academic Literature on Digital Facilitation in Primary Care

We searched the following databases: PubMed, EMBASE, CINAHL, Web of Science, and Cochrane Library. The search strategy focused on three key concepts: (1) web-based services, (2) digital facilitation, and (3) primary care settings. We restricted the searches to the European Economic Area and Organization for Economic Cooperation and Development countries as these would likely be most relevant to primary care practices in England. Full details of the search strategy are available in Multimedia Appendix 1. The first round of stage 1 searches covered publications from January 2010 to June 2020; however, these were restricted during pilot screening from January 2015 to June 2020 (see the *Study Selection* section).

#### Stage 2: Snowball Searches to Identify Literature on Digital Facilitation in Health Sectors Outside of Primary Care

In stage 2, we screened the reference lists of all articles identified for inclusion in stage 1, in addition to the reference lists of articles that we identified during stage 1 as not fitting the inclusion criteria because of article type restrictions (eg, protocols or editorials) but which were otherwise relevant.

#### Stage 3: Gray Literature on Digital Facilitation in Health Care

Gray literature was searched to identify relevant government and policy institute reports on digital facilitation in health care. This involved searches of 3 relevant not-for-profit research institutes (The Health Foundation, The King’s Fund, and The Nuffield Trust) and a health professional association website (Royal College of General Practitioners), as well as a general search of the Health Management Information Consortium database. The targeted searches of websites used combinations of terms such as *online services*, *digital*, *access*, and *patients* using Boolean operators where website search functions allowed it. The Health Management Information Consortium database allowed more complex searches; therefore, we adopted a search strategy that captured concepts related to the web (eg, web-based, digital, internet-based, and technology) and facilitation (eg, uptake, encouragement, and increased use). Full details of the gray literature search strategy are available in Multimedia Appendix 1.

#### Additional Searches Not Included in Final Review

We also explored some academic literature on digital facilitation in non–health care sectors similar to primary care in that they incorporated both web-based and offline customer services (ie, tourism and travel and retail banking) to see if any methods of digital facilitation were mentioned there that were not covered in the health care literature. These searches did not reveal additional approaches to digital facilitation and are not reported here.

### Study Selection

A key inclusion criterion for all the publications was that they addressed the facilitation of web-based services. Defining the inclusion criteria a priori was challenging, given that the key aim of this work was to define the scope of digital facilitation. We focused on web-based services that were accessed by patients through websites or phone apps facilitating access to care or providing resources for self-care and not on the delivery of medical therapies through web-based platforms, such as web-based mental health therapy. These services reflected those supported by primary care practices and were in line with the focus of NHS England at the time of this research [3]. We operationalized digital facilitation and web-based services as detailed in Textbox 1. Further eligibility criteria were tailored to the stage of the screening process (eg, primary care literature and nonprimary health care literature). Detailed inclusion and exclusion criteria for each stage of the screening process are presented in Table 1.

Before the full screening of the 11,853 publications from stage 1, we undertook a pilot screening exercise examining 237 (2%) publications, during which publications were jointly screened by 2 reviewers (EG and SP) and the results were discussed to ensure consistent approaches to screening. During the pilot screening, it was agreed that publications for stages 1 and 3 would be restricted to articles published from 2015 to 2020. For stage 2, which relied on snowball-type searches of reference lists, we included articles from 2010 to 2020 as the reference lists would have had few or no eligible articles if we did not expand the inclusion criteria to earlier years. Following the pilot screening, all remaining publications were screened independently by 1 of 2 reviewers (EG and SP).

**Operationalization of digital facilitation and web-based services.**

**Concept and inclusion and exclusion criteria**

**Inclusion criteria**

- Digital facilitation: papers that included reference to what was done to help patients access and use web-based services, including (but not limited to) the following:
  - In-person assistance with using web-based services
  - Active methods of web-based assistance for accessing services (eg, chat or help functions)
  - Passive methods of web-based assistance for accessing services (eg, frequently asked questions and help pages)
  - Telephone-based methods of providing assistance for accessing services (eg, helplines)
  - Public awareness campaigns around web-based services (if done by general practices)
  - Service improvements if done explicitly to improve or increase access
- Web-based services: web-based services accessed through a website or app, such as the following:
  - Health records
  - Prescription ordering
  - Appointment booking
  - eConsult or other web-based methods used to triage patients
  - Health care information

**Exclusion criteria**

- Digital facilitation: Papers without information on what was done to help patients access and use web-based services
- Web-based services: Non–web-based services (eg, telephone only), wearable devices, delivery of therapies (eg, psychotherapies) on the web, and web-based services for general practitioners or physicians, which did not include patients (eg, accessing continuing medical education and web-based clinical decision support tools without input from the patient)

**Table 1:** Inclusion and exclusion criteria for the screening process.

Stage of process and criteria		Include	Exclude
**All stages**			
	Scale and spread of intervention	At all scales and geographic levels from the individual site to national coverage	None
	Country	EEA^a^ countries or non-European high-income countries (defined as membership in OECD^b^)	Countries not in the EEA or OECD
	Language	English	Languages other than English
	Availability	Full-text availability	Title and abstract only and conference proceedings with no full-text article
**Stages 1 and 3**			
	Year of publication	2015 to January 2020	2014 or earlier
**Stage 2**			
	Year of publication	2010 to January 2020	2009 or earlier
**Stage 1 only: screening of academic literature on digital facilitation in primary care**			
	Topic relevance	Digital facilitation of web-based services in primary health care settings; where digital facilitation was implemented in some form: implementation as part of routine service delivery or implementation for research purposes	Where there was no reference to facilitation being implemented by or on behalf of primary care practices; thus, solely theoretical papers were excluded
	Article type	Original research	Theoretical and commentary articles; trial registrations (ie, articles registered on ClinicalTrials.gov or the WHO ICTRP^c^ registry)
**Stage 2 only: screening of literature on digital facilitation in health sectors outside of primary care**			
	Topic relevance	Digital facilitation of web-based services in non–primary care health sectors; where digital facilitation was implemented in some form: implementation as part of routine service delivery or implementation for research purposes; articles addressing aspects of digital facilitation found not to be covered by articles identified in stage 1; key gaps include evaluations of digital facilitation approaches, cost-effectiveness, and effectiveness of digital facilitation approaches for vulnerable populations	Where there was no reference to facilitation being implemented by or on behalf of health care providers; thus, solely theoretical papers were excluded; articles addressing aspects of digital facilitation already covered by the included articles identified in stage 1
	Article type	Original research	Theoretical and commentary articles and trial registrations (ie, articles registered on ClinicalTrials.gov or the WHO ICTRP registry)
**Stage 3 only: screening of gray literature on digital facilitation in health care, all sectors**			
	Topic relevance	Digital facilitation of web-based services in health care, all sectors; articles addressing aspects of digital facilitation found not to be covered by articles identified in stage 1; key gaps include the following: implications of COVID-19 pandemic for digital facilitation, evaluations of digital facilitation approaches, and effectiveness of digital facilitation approaches for vulnerable populations	Where there was no reference to facilitation being implemented by or on behalf of health care providers; thus, solely theoretical papers were excluded; articles addressing aspects of digital facilitation already covered by the included articles identified in stage 1
	Article type	Gray literature (ie, literature produced in electronic and print formats outside of commercial publishing), including but not limited to government documents or reports, policy reports, research reports, and working papers	Trial registrations (ie, articles registered on ClinicalTrials.gov or the WHO ICTRP registry)
	Article type	Original research	Theoretical and commentary articles and trial registrations (ie, articles registered on ClinicalTrials.gov or the WHO ICTRP registry)

^a^EEA: European Economic Area.

^b^OECD: Organization for Economic Cooperation and Development.

^c^WHO ICTRP: World Health Organization International Clinical Trials Registry Platform.

### Data Extraction and Preliminary Analysis

Data from eligible studies were extracted independently by 2 reviewers (EG and SP) using a data-charting form developed for this study. The form was piloted to ensure that data extraction was consistent across reviewers. We extracted data relevant to digital facilitation (digital technology type, facilitation purpose, method, mode of delivery, target population, and setting) and study details (study type, outcomes, size, and setting), aiming to capture health outcomes, staff and patient or caregiver experience, impact on service use (uptake and use of digital services), cost and equity of access to health care services and information, and the nature and extent of other reported outcomes. When considering the outcomes of digital facilitation, we focused on increased uptake and use of web-based services by patients, defining these as indicators of successful facilitation.

Studies were not formally assessed for quality as this was a scoping review, with a great breadth of studies and article types being included. However, reviewers noted the quality of the evidence source, clarity of aims, quality and comprehensiveness of the work, and any conflicts of interest from the authors wherever possible to assist in judging the quality of the overall evidence base for digital facilitation. Given that we did not formally assess the quality of individual studies, we did not report on study quality. Full details of data extraction are available in Multimedia Appendix 2.

### Data Analysis, Synthesis, and Typology Development

Preliminary analysis of the data extracted from stage 1 was undertaken to identify gaps in the literature and to inform subsequent stages (Figure 1). Following all extractions, data analysis followed the principles of narrative descriptive synthesis [29]. Key themes were identified and captured during charting, which were then refined and expanded during the preliminary synthesis. The synthesis involved an iterative process of internal study team discussions, analyses, and writing. The typology of digital facilitation approaches was developed through this process of internal team discussion and the synthesis of evidence. Further refinement of themes, initial synthesis, and typology was undertaken through a workshop with study team members, including patient and public involvement representatives.

## Results

### Overview

**Figure 2:**
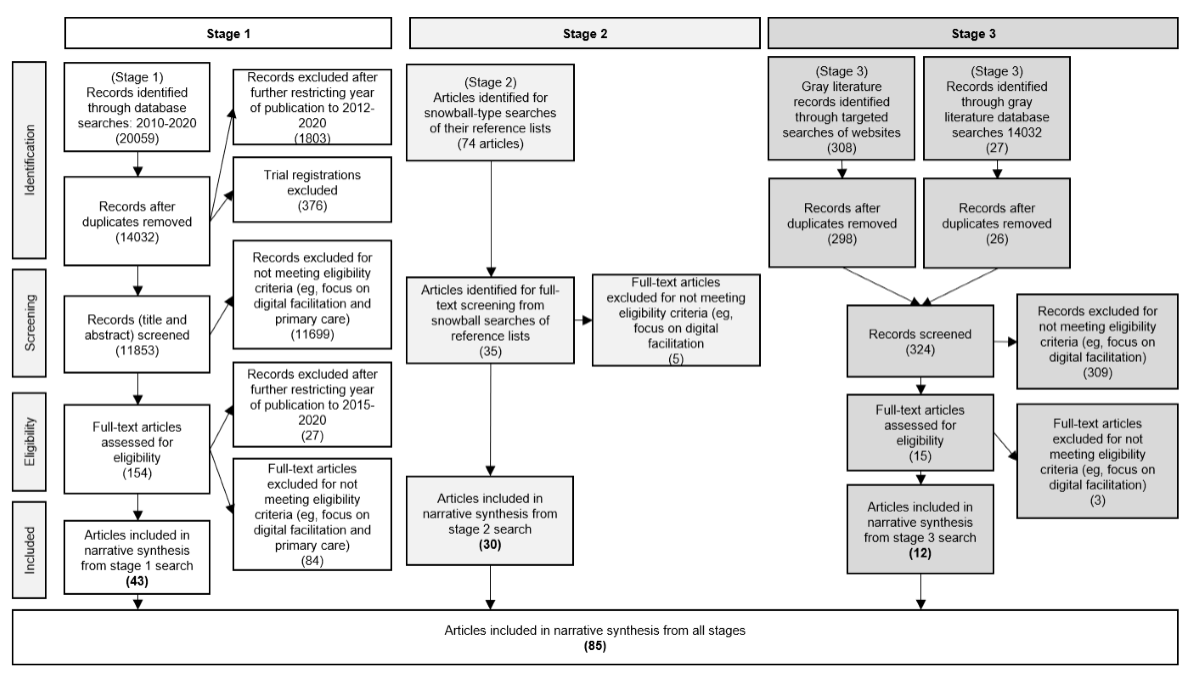
PRISMA (Preferred Reporting Item For Systematic Review And Meta-Analyses) flow diagram of literature review.

The PRISMA (Preferred Reporting Item for Systematic Reviews and Meta-Analyses) flow diagram (Figure 2) shows the number of publications retrieved and excluded at each stage. In stage 1, a total of 11,853 records were screened, of which 43 (0.36%) met the inclusion criteria. Later stages identified an additional 42 publications for a total of 85 full-text publications that were included. Multimedia Appendix 3 [6,21,30-105] shows a full list of the included publications and their characteristics.

Publications focused on the United States (30/85, 35%), the United Kingdom (19/85, 22%), other European countries (23/85, 27%), Australia (8/85, 9%), or Canada (1/85, 1%) or adopted an international focus (5/85, 6%). They covered digital facilitation in the primary care sector (48/85, 56%), secondary care sector (5/85, 6%), tertiary care sector (1/85, 1%), or all health care sectors (nonspecific; 31/85, 36%). The publications used various study designs, including quantitative approaches (37/85, 44%; randomized controlled trials [RCTs]: 15/85, 18%; prospective cohort: 4/85, 5%; retrospective cohort design: 1/85, 1%; retrospective observational: 1/85, 1%; longitudinal observational: 1/85, 1%; cross-sectional: 9/85, 11%; pre-post analysis: 1/85, 1%; secondary analysis of data from RCTs: 3/85, 4%), mixed methods approaches (7/85, 8%), qualitative approaches (31/85, 36%), and literature reviews (12/85, 14%). Publications focused on a variety of disease areas, with the most common being diabetes (12/85, 14%) and depression (10/85, 12%).

### Typology of Digital Facilitation

#### Overview

A wide variety of digital facilitation efforts were discussed in the literature. In our proposed typology, we categorized them according to whether they were aimed at patients or staff and the purpose of facilitation within them (Table 2). In the following sections, we summarize the descriptive accounts of the different types of digital facilitation and synthesize evidence on whether the approach appeared to be associated with the initial uptake and subsequent use of digital services where available.

**Table 2:** Typology of digital facilitation approaches.

Typology of digital facilitation			Definition		Examples of facilitation approaches
**Digital facilitation aimed at patients**					
	Promotion	Broad category of digital facilitation that captures ways of raising awareness of and knowledge about digital services, endorsements of specific digital services to patients, and methods of encouraging patients to use them		Recommendation and prescription of digital services and other communication-centered interventions; emails and written reminders; video introductions to digital services	
	Training and education	Education or training to help patients acquire technical skills to use digital services or to help patients understand what features of a digital service can be most helpful to them		Initial assistance with the use of digital services	
	Guidance and support	Ongoing help in using digital services provided by clinicians or other primary care staff to patients		Coaching and ongoing guidance from clinicians and other staff	
Digital facilitation aimed at primary care staff			Interventions aimed at primary care staff, typically to increase staff’s knowledge of digital services so that they can better support patients in their use of the services or to increase their trust in services to increase the likelihood of staff promoting the service to patients		Certified list of apps and websites (to be able to recommend to patients); practice champions (to increase buy-in); training for providers (to generate awareness of web-based services and how to use them)

#### Digital Facilitation Aimed at Patients

Most (71/85, 84%) articles reported on facilitation efforts aimed directly at patients as distinct from supporting health care staff in helping patients. Facilitation aimed at patients was grouped into three categories: (1) promotion, (2) training, and (3) guidance and support.

##### Promotion

A lack of knowledge by patients of available web-based services is a significant barrier that primary care staff can help to overcome [30]. The evidence suggests that promotion is a broad category of digital facilitation referring to ways of raising awareness of, as well as knowledge about, digital services; providers endorsing specific digital services to patients; and encouraging patients to use them. Similarly, promotion can take place across a range of media, including on the web, in person during appointments [31], and in less personalized forms such as placing posters or promotional material in waiting rooms [32].

Examples of web-based promotions include practices featuring links on their websites to promote eConsult (e-consultation and self-help web service [33]), sending reminders or links to web-based services via email or SMS text messages, and using web-based promotional videos. Engaging patients by providing an electronic device such as a tablet for use in the practice waiting room rather than simply relying on verbal recommendations has also been explored in a feasibility study as a way of motivating patients to continue using a web-based self-regulation program once they return home [34]. Verbal recommendation by staff is one of the most widespread, routinely used methods of digital facilitation [35-38].

##### Training and Education

Training and education may also promote the uptake and use of digital services, both by helping patients acquire technical skills to use web-based services and by helping them understand what features of a web-based service can be most helpful [39]. In the literature we reviewed, training was delivered on the web through videos [40] or offline through in-person support [41,42], presentations, or seminars [43] and was delivered either in a single session [40] or over several sessions [43]. Training and education were commonly combined with helping patients initially sign up for a specific web-based service, such as a patient portal [41,42].

Examples of training included community health care workers conducting home visits to help patients use web-based portals [44]; an in-person tutorial delivered by mental health experts, research assistants, and research nurses within primary care offices [45,106,107]; and a peer support specialist with personal experience in mental illness, substance use, or behavioral concerns to provide technical support to patients using an app within the veteran health system in the United States [46]. There were also fewer resource-intensive facilitation efforts described in the literature, for example, through combinations of written materials, videos, and oral communication about how to use patient portals [41,47].

Training and education delivered through videos were also common [45] and could vary from a short 7-minute video demonstrating how a digital service could be used to a series of 11 videos about how to use patient portals, each with a different theme (eg, patient stories, getting started, signing up and creating a username, accessing different services within the app, and showing patients how to message providers) [106].

Some training and education focused on specific digital services and on providing patients with information on how to use them, whereas other interventions were more general and oriented toward digital literacy and digital health literacy. For example, several qualitative studies recommended that providers suggest computer classes [48], particularly to older adults and patients from minority ethnic groups, to help them use web-based information [49-51]; another study found that digital health literacy courses would be helpful for patients with chronic obstructive pulmonary disease [52].

##### Guidance and Support

Guidance and support refer to ongoing help provided by clinicians or other primary care staff. It focuses on the technical aspects of using digital services, similar to training, but appears to often focus on interventions that help patients set goals, keep track of progress, and improve adherence and other less technical aspects of digital services. Guidance and support may be particularly important, as a lack of trust and communication has been associated with the discontinued use of web-based platforms [53]. Ongoing guidance and support may be provided through in-person meetings, phone calls, and home visits or in other settings.

*Practice champions* [108] have been used in primary care to increase the use of web-based services. As experts in a particular web-based service, they provide assistance and ongoing support to patients with the potential to increase both initial uptake and continued use of web-based services thereafter.

#### Digital Facilitation Aimed at Primary Care Staff

For primary care staff to be able to help patients use web-based services, they must first be aware of what services are available, how they work, why they are useful and trustworthy, and how they can benefit specific patient groups [54]. Health care professionals also need to be clear about their role in terms of endorsing and facilitating web-based services [55]. There is evidence that some GPs are opposed to the use of web-based services by patients [56]. This can include GPs believing that web-based services generate additional workload or preferring to have patients engage directly with the GP [56]. Partly for these reasons, efforts have been made to train primary care staff and increase their knowledge, understanding, and confidence in web-based services.

Some digital facilitation efforts aimed at staff involve interventions to encourage GP practices to adopt more web-based services and actively promote them to their patients. In the United Kingdom, researchers held practice-level discussions with GPs to tackle the strong views held by some GPs against prescribing web-based information, albeit with limited effect [56]. In Spain, an experimental study provided physicians with a list of mobile apps that had been certified by public health authorities and examined the effects of physicians prescribing the apps on patient uptake and use of digital services. As staff buy-in is an important enabler of digital facilitation, having a list of trusted apps can be valuable [57].

Other facilitation efforts focus on training health care practitioners, as studies suggest that staff need training to acquire the necessary technical skills to use web-based services [58] or to effectively reach the target population [59,60]. For example, staff may be trained in communication strategies and relationship building, so that patients or their families are more likely to follow advice to use digital services [59,60]. Such training may be delivered through web-based meetings, face-to-face sessions, and presentations or by sending explanatory videos to the staff [61].

### Potential Disadvantages and Risks

Although there is a wealth of literature on the potential harms of digital services, including in terms of health and digital inequalities, there is less information in the literature included in this review discussing the potential disadvantages of *facilitation efforts* specifically. An example in the included literature was that communication-based facilitation efforts that require high levels of emotional engagement may contribute to distress and fatigue among staff [59]. Another example is that lists of approved apps risk being biased in the considered sample of apps when the onus is on app developers to apply for inclusion in the lists [62]. Some patients have concerns about whether web-based support to encourage continued engagement with digital services would replace valued in-person contact [53]. Email reminders, although sometimes useful, can also cause patients to avoid certain web-based services so as not to receive reminders, although this depends on individual preferences around the frequency of reminders [63]. Finally, the facilitation that provides patients with tablets or computers to use digital services in waiting rooms may compromise patient confidentiality [64].

Evidence also suggests that health care staffs’ perceptions of harm from digital services, such as negative impacts on the patient-provider relationships, increased workload, and patients misinterpreting web-based health information, may negatively affect their willingness to recommend digital services to patients [65].

### Association of Digital Facilitation With an Increase in Uptake and Use of Digital Services

#### Overview

The evidence relating to whether different digital facilitation approaches increase the uptake and use of digital services is summarized in Table 3 and is described in the following sections.

**Table 3:** Evidence on increasing uptake and use of types of digital facilitation approaches.

Typology and digital facilitation effort			Evidence for increasing uptake and use
**Promotion**			
	Recommendation or prescription of digital service to patient	Staff recommendation or endorsement of a digital service was shown to be one of the most effective ways of increasing patient uptake and use in 2 literature reviews on the topic [21,66]. Qualitative evidence from primary studies also supports staff recommendation or endorsement as an effective way of boosting the use of digital services [35,67,68]. There is strong evidence from RCTs^a^ supporting that prescription and referral pads for digital services are effective in increasing patient uptake [61,69], along with evidence from a review on the topic [70]. There is some evidence that a list of certified apps and websites (approved by a regulating body) may be effective in enabling providers to prescribe apps and websites to patients [57]. However, when it was implemented by the NHS^b^, it had a lack of brand recognition and was ineffective in encouraging the use of high-quality web-based services [71]. Multiple mixed methods studies suggest that recommendation or endorsement of digital services may be more effective when staff focus on specific aspects of digital service, which will be useful to particular patients, and gradually introduce patients to digital services based on their individual needs at that time [52,65,71-73].	
	Communication-centered interventions	Qualitative evidence and evidence from an RCT suggest that recommendation or endorsement of digital services may be more effective when staff are trained in how to best engage patients using specific communication strategies and shared messaging around the service [33,52,53,59,60,64]. For example, these communication strategies can include motivational interviewing and “ICE” formats to address patient ideas, concerns, and expectations. There is strong evidence from 3 RCTs that interventions that help patients form specific “if-then” plans to use digital services are effective in increasing the continued use of digital services [74].	
	Email and written reminders	Mixed methods and qualitative studies have shown that written materials such as brochures, leaflets, and advertisements may be effective in increasing patient use of digital services and are useful in that they require little effort from providers [33,65,75]. Reminders (eg, SMS text messages and push notifications) have been implemented in some areas [64,76], and feedback from patients and service users suggests that they may be useful in increasing uptake and use [48,53].	
	Video introductions to digital services	There is mixed evidence from RCTs on whether video introductions are effective in increasing the uptake of digital services. There is no evidence to support they are effective in increasing the sustained use of digital services [40,47,77,106].	
	Public information campaigns	In the United Kingdom, a public information campaign and personalized invitations to invite patients to use an electronic health record system were found to be ineffective in encouraging enrollment [32].	
**Training**			
	Initial assistance with use of digital services	There is mixed evidence from RCTs and quantitative studies on whether initial assistance in registering and logging into digital services is effective in increasing uptake and use [41-43,45]. There is qualitative evidence suggesting patients and providers feel this type of assistance would be useful [78,79]; however, the weight of the evidence suggests that it is likely ineffective and that additional continued support is needed to encourage continued use of digital services. There is qualitative evidence suggesting that allowing patients to log into and use digital services in primary care practices (eg, in the waiting room on tablets) may be effective in encouraging patients to continue using a service outside of the practice [34,80]. This intervention has been implemented in studies with some success [64].	
	Technical training support	There is a body of literature (including strong evidence from a systematic review and an RCT) emphasizing the importance of technical support for using digital services and wider support for digital literacy and digital health literacy in encouraging patient use of digital services, [31,48,51,52,106,109], particularly for older patients, patients from ethnic and racial minority groups, and patients in low-income settings. However, at least one RCT found that simply providing information on using the internet was not effective in increasing the use of digital health services [56].	
**Guidance and support**			
	Coaching and ongoing guidance for patients	There is mixed evidence from RCTs and nonrandomized trials on whether ongoing coaching and support is broadly effective in increasing uptake and sustained use of digital services [44,107,110-112]. The weight of evidence suggests that certain forms of ongoing support are likely effective (see the following sections). There is strong evidence from 3 RCTs suggesting that ongoing guidance focused on adherence, content of digital services and goal setting are likely more effective than ongoing guidance on only technical aspects of digital services in increasing the use of digital services [74], which is also supported by qualitative evidence [78]. Qualitative evidence suggests that both face-to-face and telephone support is likely important in encouraging patients to continue to use digital services [53,57,63,109,113].	

^a^RCT: randomized controlled trial.

^b^NHS: National Health Service.

#### Promotion

##### Recommendation and Prescription of Digital Services and Other Communication-Centered Interventions

Evidence suggests that promotion increases the initial uptake and subsequent use of digital services [66]. A review of promotion methods suggested that endorsement by health care staff is one of the most influential factors affecting patient uptake and use of patient portals [21]. Qualitative findings suggest that recommending digital services to patients is effective in increasing the uptake of those services [35,67,68] where staff focus on specific features of a digital service that will be useful to individual patients [37,52,65], staff are trained in how to best engage patients [60], and staff have a shared understanding of the messaging around digital services [33,64]. Written prescription or referral pads to *prescribe* digital services have also been shown to increase patient uptake of digital services [61,70]. However, a quasi-RCT in the United Kingdom found that providing patients with booklets with general information about using the internet for health purposes was ineffective in increasing their readiness to use electronic health services [56].

Certain communication strategies have been shown to increase the uptake of digital services, such as relationship-building techniques [59]; interviewing and conversational techniques such as motivational interviewing [53]; and discussing patients’ ideas, concerns, and expectations to help address patients’ misconceptions [52]. Evidence also suggests that gradually introducing patients to digital services, or introducing new features, over the course of several visits rather than all at once can improve the uptake and use of digital services [72,73]. Helping patients form specific plans around the use of digital services was shown to be one of the strongest predictors of adherence in an RCT of an internet-based intervention for depression [74].

##### Emails and Written Reminders

The written material that health care staff can provide to patients about digital services may be useful in encouraging uptake, incurring minimal time and effort from the staff [65,75]. In a UK study where e-consultation and self-help web services were promoted through posters, leaflets, and advertisements on television screens in waiting rooms and on practice websites, 79% of those who used the web service reported that they discovered the service through these promotion efforts [33]. Reminders for participants can also be helpful in encouraging the uptake and use of digital services [48,53], for example, through SMS text messages sent by receptionists with links to web-based tools [64] or sent to patients at key times, such as when health care staff upload new notes to patient portals, which in one quasi-experimental study resulted in >85% of patients viewing at least one note on the patient portal [76]. However, an RCT examining adherence to an internet-based therapy program for depressive symptoms among high school students found that neither tailored nor standardized emails increased adherence in this group [81].

#### Training and Education

##### Initial Assistance With and Education on Use of Digital Services

The evidence is mixed about whether initial assistance with, and education on, the use of digital services increases uptake and continued use. There are contradictory findings on whether initial assistance and education increase the initial uptake or sustained use of digital services.

A quantitative study of the uptake and use of patient portals for patients with chronic kidney disease found that renal clinics that helped patients with initial log-in and registration to the portal had higher levels of portal uptake and use than clinics that did not, with patients 20% more likely to be continued users of the portal after 3 years [42]. A study based on interviews with providers suggests that letting patients use tablet devices or computers in practice waiting rooms may encourage their later use at home [34]. Both health care staff and patients expressed enthusiasm about the potential to access health information [80] and complete digital screening tests [64] on tablets while waiting for appointments.

Although evidence from these studies suggests that the impact of some education and assistance sessions may be long lasting, there is contradicting evidence from other studies indicating that initial training or introductory educational sessions have little impact on use after the initial sign-up [41,43,66]. For example, a study entailed clinical staff providing a 10-minute training session to prospective patient portal users on using and installing a phone app to access the portal, including troubleshooting issues during the training session and providing a pamphlet with further information on the patient portal. It found that although patient interest in the app was high, actual portal use did not increase after the intervention [41]. Similarly, an RCT regarding the effectiveness of an initial 10-minute standardized personal information session on internet-based depression interventions found that these sessions were ineffective in increasing adherence in an inpatient and outpatient rehabilitation setting for diabetes care [45]. Furthermore, an RCT from the Netherlands showed that initial group education sessions for patients with type 2 diabetes to help them set goals and use web-based platforms were ineffective in increasing the use of the service [43].

##### Video Introductions to Digital Services

Approximately 5% (4/85) of RCTs evaluated the effect of video introductions on patient uptake and use of digital services, with mixed findings suggesting that video introductions may increase initial uptake but are unlikely to contribute to sustained use. Although 2% (2/85) of studies found that web-based video-based training increased patient *uptake* [47,106], one of the studies found that only 3.5% of patients who were given a video introduction continued to use a portal compared with 1.2% of those who received paper instructions and 0.75% of those who received no intervention, indicating *low sustained use* for all patients [47]. A third RCT found that a 3-minute video was not effective in increasing the uptake or use of a web-based intervention for chronic pain [77]. Another RCT found that a 7-minute video was effective in increasing acceptance of internet-based interventions for depression, although actual use was not measured [40].

#### Guidance and Support

There is evidence that ongoing support from clinicians and other staff members can increase the use of digital services, although some studies have found these interventions to be ineffective.

Several RCTs have compared the effectiveness of clinicians or other staff in guiding patients in the use of digital services with self-directed services. One of the studies found that patients using web-based therapy for chronic pain who were guided by a psychologist completed more modules than unguided groups and had lower attrition rates [110]. A series of RCTs in Germany comparing different forms of ongoing guidance from clinicians and other staff members assessed how they influenced adherence to digital interventions. The analysis found that both content-focused (personalized written feedback from a psychologist–health coach and reminders to complete modules) and adherence-focused guidance (reminders to complete modules and ability to request feedback from a psychologist–health coach) were equally effective in increasing adherence compared with administrative guidance (technical support in case of computer and internet issues) [82]. However, 2% (2/85) of other RCTs examining the effect of guides on the completion of web-based modules [111] or patient portal use [44] showed either mixed or no evidence for the effectiveness of guides.

Several quantitative studies with nonrandomized control groups also tested the effectiveness of guides in helping patients engage with app content. Some interventions, such as sessions with health coaches [112], hands-on and telephone assistance from nurses, and an intensive course for patients [107], may increase the uptake and use of digital services.

Qualitative evidence also suggests that face-to-face support for patients along with ongoing web support may facilitate the uptake and use of digital services [57,63,109,113]. For example, incorporating digital services into regular care and providing patients with a way of messaging providers for support may encourage sustained engagement [53]. In addition, ongoing training in the use of particular digital services or, more generally, to increase digital literacy skills may encourage uptake and use [48,52,109].

### Evidence Relating to Inequality Between Different Population Groups

We found little evidence from studies examining digital facilitation for vulnerable populations, and no studies directly compared different approaches. However, few studies identified strategies that may be effective in increasing the uptake and use of digital services in specific patient populations. For example, a systematic review found that technical training and assistance programs have the best evidence for increasing portal use for vulnerable populations (older adults; racial minorities; and individuals with low socioeconomic status, low health literacy, chronic illness, or disabilities) and that other interventions do not have sufficient evidence [31]. A US study found qualitative evidence that ongoing training, both in the use of a particular service and more generally to increase digital and health literacy skills, can help address the barriers to receiving care faced by African American and Latino patients [50] and patients in low-income areas [51].

There is concern that older people will need extra support to be able to use digital services [66,71]. Some studies showed that older patients were more likely to use digital services after facilitation efforts [106]. Ongoing training and support may also be helpful in encouraging the uptake and use of digital services among older people [48]. Despite concerns about older adult groups being less able or willing to use technology [55,67], evidence suggests that they are nevertheless often willing to use tablets [80], patient portals [21], remote video consultations [73], and health-related apps [62]. Several studies pointed to the importance of ongoing human support [53] and training on both the technical aspects of digital services and general digital literacy skills for older patients [48]. Several studies included subgroup analyses, which revealed that patients with lower health literacy or disabilities were less likely than others to use digital services even after facilitation efforts [43,66,106].

There is some evidence that providers may be more willing and able to engage in digital facilitation efforts with patients who are already confident users of digital services, including the *worried well*, potentially exacerbating inequalities in access to digital health resources [71,83]. A review found that providers are more likely to recommend digital services to patients they perceive as more technologically knowledgeable, and these perceptions may be based on age, socioeconomic status, education level, and ethnic group [36,65].

### Factors Affecting Successful Digital Facilitation

#### Perceptions of Usefulness of the Digital Service

One of the most commonly reported factors influencing the success of digital facilitation efforts in primary (and secondary) care settings is the perception, both from the patients and the health care staff, that digital services will be useful [38,39,55,57,58,72]. Patients are more likely to use services that have been recommended by health care staff if they see the information and functionality as novel [32], if they are able to customize the service to their own needs and preferences [21,63], and if the service is sufficiently specific to fit their needs [84]. Qualitative evidence suggests that the health care staff’s likelihood of recommending a digital service to patients may be influenced by the alignment of information within apps and websites with the health information and recommendations that physicians commonly provide to patients [39] and by the availability of evidence that digital services result in patient benefits [30,52].

#### Time and Capacity in Primary Care

Challenges in terms of staff having sufficient time to implement digital facilitation efforts were commonly identified in the literature [30,34,35,37,46,57,59,64,73,85,113]. The literature also indicated ways of helping to address this issue. Email templates, protocols, and scripts can help staff automate some aspects of patient engagement [70]. Passive facilitation efforts such as posters and brochures can also help mitigate time pressures in primary care [75]. In some studies, it was found to be helpful to have staff other than physicians engage with patients in digital facilitation efforts because of time constraints for physicians [37,64,75,78] or to use the time that patients spend in waiting rooms as an opportunity to facilitate access to web-based services [34,64,80]. Limited time during GP consultations may make it difficult to engage patients in digital facilitation efforts [64]. One of the studies suggested that facilitation efforts may be more feasible during certain kinds of appointments where patients may have less pressing concerns (eg, vaccination-, contraception-, nutritional-, and physical activity–focused appointments) [34].

#### Buy-in From Health Care Staff

Staff buy-in and motivation were important enablers in many of the studies [42,59,62,86-88], and negative staff attitudes or a lack of motivation toward an intervention were often barriers to facilitation efforts [56,57,60,85]. In several studies, staff buy-in was encouraged through early engagement of staff when developing an intervention, initial education, or training sessions in practices to introduce staff to new web-based services or interventions, ongoing communication with staff, and incorporation of digital services into discussions at staff meetings [30,35,54,60,78]. Ongoing education and training for health care staff on how to use digital services have also been indicated as important in helping them engage in digital facilitation [52,65,70,89].

Reshaping roles in the NHS to incorporate digital services and digital facilitation may also be important in securing staff buy-in [66,73,90]. This not only applies to GPs and nurses but also to wider primary care support teams. Seeing digital facilitation as part of their role rather than something added to their existing job was important in increasing acceptance and buy-in among practice receptionists in a study that required them to send reminders to patients [64].

#### Patients’ and Staff’s Trust in, and Knowledge of, Digital Services

Qualitative studies have shown that patients’ lack of trust in web-based services can be a barrier to using them [50,80,91], and this is an issue reported by older patients in particular [49]. Fears of loss of confidentiality and security of web-based information may also affect the staff’s willingness to recommend digital services to patients [50,65]. Efforts to increase the perceived security of websites were described in the literature, such as the use of third-party seals on patient portal websites [50].

#### Guidelines and the Role of Regulators

The existence of guidelines that help providers recommend digital services to patients may also be helpful in facilitating efforts [73,87]. Evidence from qualitative studies highlights the importance of simple recruitment criteria, referral guides, and specific triggers that prompt the recommendation of digital services to patients [49,54,60,61,70]. In some cases, mandates for recommending services have also been helpful [89]. In the United Kingdom, it has been suggested that setting targets for GPs to encourage the use of digital services could potentially be effective in increasing patient uptake [66]. Policies that make funding available for training, organizational development, and infrastructure, as well as technology that allows providers to facilitate the use and uptake of digital services, will also be important in increasing use among patients [62,66,73,87,90].

#### Patients’ Trust in Health Care Staff

Trust, perhaps promoted by long-term relationships with health care staff, may be important in patients’ use of digital services recommended by those staff [67,114]. Where providers give ongoing support to patients in using a digital service, trusting relationships and a positive, personal tone may boost patients’ motivation to participate in digital interventions [53].

## Discussion

### Typology of Digital Facilitation

We found a rich vein of information about ways in which health care staff in primary care settings can facilitate patients’ use of digital services. There is a wide range of approaches to digital facilitation. On the basis of the literature, we developed a novel typology encompassing digital facilitation aimed at patients (promotions, training and education, guidance, and support) and digital facilitation aimed at primary care staff to facilitate patients’ use of digital services.

Our review shows diversity in the types of interventions that can be considered under the umbrella term *digital facilitation*. Developing a common framework to define and categorize these advances the evidence base and informs the selection and implementation of different types of facilitation. It also furthers the conceptual understanding of digital facilitation, which is important for informing evaluations of facilitation approaches. Understanding which approaches work best for whom and in which contexts will be critical for enabling widespread equitable use of digital technologies in primary care, and developing an accurate typology is a necessary first step. We anticipate that future research will seek to refine our proposed typology further. From our review, possible areas for further differentiation may relate to the mode of delivery (eg, in person or on the web) and the degree to which facilitation is passive or active.

### Effectiveness of Digital Facilitation Efforts

Our review found evidence that most digital facilitation efforts can support the initial uptake of digital services by patients but that they are unlikely to contribute to sustained use. For example, we found evidence that promotion efforts such as recommendation by practice staff, prescription of digital services, or email and written reminders may increase initial uptake; however, there is little evidence that they lead to sustained use. Similarly, training and education on the use of digital services, such as providing initial assistance with registering for services, also appears to encourage the initial uptake of digital services; however, evidence suggests that these efforts are insufficient to promote long-term use.

Hands-on facilitation approaches, including promotion, guidance, and support by staff, have provided some of the most consistently positive evidence of the usefulness and may be especially important for older adults. This has resource implications as guidance and support take time, and active facilitation takes more time. However, no study has yet examined the cost-effectiveness of digital facilitation. Current mandates in England incentivize the uptake of services and encourage primary care practices to promote web-based services to patients through recommended methods such as posters in physical practices, promotions on practice websites, verbal promotion by practice staff, and promotion via email [3]. However, as identified in our review, promotional approaches may increase initial uptake but seem to not contribute to the sustained use of digital services. It may be that mandates and recommended approaches to digital facilitation need to be revised to recommend approaches such as guidance and coaching, which also incentivize more sustained use. However, without adequate evaluations, including cost-effectiveness studies, it is unclear whether such a mandate is warranted.

### Facilitators and Challenges Associated With Digital Facilitation Efforts

Several factors may increase the success of digital facilitation efforts, starting with perceptions among staff and patients that the digital service in question is useful. However, with so many digital services available, it can be challenging for practices to identify appropriate and effective services [115]. Therefore, an important precursor to effective digital facilitation is supporting evaluations of available digital services to help practices and local health authorities understand their impact, affordability, sustainability, and scalability [116]. This would allow practices to prioritize the services likely to be most useful in their local context and has the potential to enhance the trust of health care staff in specific digital services because of their increased knowledge about the services, both of which were found to contribute to the success of facilitation efforts.

Given the current workload pressures on primary care physicians and staff [117-119], actions that reduce the time required to provide facilitation, such as providing guidelines to help practices determine which digital services to prioritize, could increase the success of facilitation efforts. Furthermore, such approaches could also help practices meet the broader aims of digital primary care in the NHS to improve the quality of care to patients and provide efficiency gains to practices [120]. Primary care providers need to feel that the incorporation of patient-facing digital services into their practices is a net gain in terms of workload and efficiency, including any time spent on digital facilitation; otherwise, they may resist efforts to make more services available on the web.

Our review also showed that patients may be more likely to take up and continue to use digital services endorsed or recommended by GPs or other health care staff who they trust. Understanding the value and limitations of these trust relationships is especially important for ensuring the equitable uptake of web-based services. There is substantial evidence that ethnic minority groups in the United Kingdom have lower levels of trust in health care providers and, as a result, face barriers to care [121]. Communication strategies focusing on building trust and positive relationships can help increase the effectiveness of digital facilitation efforts. When positive trust-based relationships exist between GPs and patients, our research suggests that this relationship can be effectively leveraged to help patients access digital services. However, if used in isolation, this approach may leave already disenfranchised groups further excluded from valuable health care resources.

Any implementation of digital facilitation should consider its potential risks and disadvantages, however, this review found little information in the literature specifically discussing the potential disadvantages of facilitation efforts. The available evidence suggests the potential for facilitation efforts to contribute to distress or fatigue among staff. Although this review focused specifically on facilitation efforts, it is reasonable to assume that the harms of digital services being promoted to patients through these facilitation efforts would be important to consider in terms of the risks and disadvantages of facilitation. For example, there are concerns from patients and providers that digital services may replace valued in-person contact or interfere with patient-provider relationships. Holding negative views about web-based services may decrease patients’ or providers’ willingness to engage in digital facilitation efforts. It would also be important to consider the cost-effectiveness and opportunity costs in terms of primary care staff spending time and resources on digital facilitation rather than other activities.

### Agenda for Future Research

Given the push by the NHS for primary care practices to move services to the web [3,5] and the increase in patients’ use of web-based services [122], the lack of evidence on how best to facilitate patient access to these services represents a significant gap that should be addressed through future research. A valuable next step would be in-depth qualitative studies that refine our understanding of how digital facilitation occurs in practice, including identifying where its boundaries lie and how staff and patients engage with facilitation efforts.

Future research should also evaluate the effectiveness and cost-effectiveness of digital facilitation interventions. These studies should focus on outcomes such as the impact on service provision or service use more broadly, as well as on the impacts on patient and staff satisfaction, aspects that were absent in the literature. Future research should also consider the potential unintended impacts of digital facilitation, such as increased inequities in access. Furthermore, limited evidence is available to inform the routine use of digital facilitation in primary care. Consideration of a wider range of outcomes, including patient benefits and costs from service, staff, and patient and caregiver perspectives, will help inform decisions about digital facilitation in primary care practices. Evidence suggests that the context of the patient group, existing relationships, and trust in services can all be important considerations in the effectiveness of facilitation efforts [49,50,53,67,80,91,114]. Understanding this further in the design and evaluation of digital facilitation is important. Our focus on digital facilitation underpinning the organizational aspects of primary care service delivery rather than on exploring the facilitation of digitally delivered therapeutic interventions is a limitation we recognize and which would be a fruitful area for future research.

Only a few studies identified strategies that may be effective in increasing the uptake and use of digital services in specific patient populations. These include technical training and assistance programs for vulnerable populations and providing human support for older adults. Given the existing evidence of inequalities in access to web-based services [15,16] and the role of primary care as the first point of contact for most people [26], this is likely to need careful consideration as NHS England moves forward with its Digital First approach [27]. Evidence on targeting interventions for different groups of patients and for different types of web-based services should be prioritized.

As noted, the COVID-19 pandemic has had an impact on the uptake of digital services [123]; however, it is not clear how digital facilitation efforts have been affected. At the time of our review, no published evidence was available on the impact of COVID-19 on digital facilitation efforts. Timely research is required to more fully understand the pandemic’s impact on the provision of digital services in primary care and, crucially, how practices facilitate access, particularly to vulnerable groups and those in most need of support. It is easy to think that digital facilitation may be less important, given that the pandemic has led to a surge in the use of digital services; however, as others argue, ensuring that increases in uptake are sustained will be crucial, and in the context of disrupted and backlogged routine care, digital services are likely to become increasingly important [6].

### Strengths and Limitations

As a scoping review, a formal quality assessment of studies was not undertaken, which limited the assessment of the strength of evidence in this review. However, this allowed us to capture the breadth of the literature on digital facilitation in primary care. We were able to describe the breadth of the types of facilitation and provide some assessments of usefulness based on diverse evidence. It is possible that in restricting the selection of publications to 2015 onward, we may have missed earlier publications of relevance; however, from our staged and iterative process in restricting the date, we do not anticipate that this was likely.

### Conclusions

The number of drivers to increase the use of digital services in primary care is likely to increase. Digital facilitation is a useful umbrella term that we have introduced into the literature to describe a range of efforts seeking to promote the uptake and use of digital services. Our review found diverse examples of digital facilitation targeting either patients or health care staff. Evidence of its effectiveness was limited, with no evidence of cost-effectiveness. Methods of promotion or initial training appear to be effective in increasing the initial uptake of services but not sustained use without further support. Incentives or requirements for practices to increase the uptake of digital services should also include ongoing use. Despite growing concerns about inequalities in the uptake of digital services, there is limited consideration of this in the literature, either in the design or evaluation of interventions. There is a need to improve both the conceptual understanding and evaluations of digital facilitation. This study offers an initial typology that helps inform both of these key areas of consideration.

## Appendix

Multimedia Appendix 1 Details of literature searches.

Multimedia Appendix 2 Data fields in data extraction chart.

Multimedia Appendix 3 Full list of publications included scoping review of the literature.

## Abbreviations

GP: general practitioner
NHS: National Health Service
PRISMA: Preferred Reporting Item For Systematic Reviews And Meta-Analyses
PROSPERO: International Prospective Register of Systematic Reviews
RCT: randomized controlled trial
