# Supplementary material for: Digital Facilitation to Support Patient Access to Web-Based Primary Care Services: Scoping Literature Review
Source: J Med Internet Res. 2022 Jul 14;24(7):e33911. doi: 10.2196/33911 (PMC9335178; doi:10.2196/33911)
Supplement: Multimedia Appendix 1 [file jmir_v24i7e33911_app1.docx]

# Supplement A. Details of literature searches

# Stage 1 searches of the academic literature on digital facilitation in primary care

## PubMed

| **Limits: English; 2010-present Search run: 18 June 2020** |
| --- |
| ((online[tw] OR on-line[tw] OR web[tw] OR website[tw] OR internet[tw] OR network[tw] OR digital[tw] OR smartphone*[tw] or "smart phone*"[tw] OR app[tw] OR computer*[tw] OR "mobile phone*"[tw]) AND (consult*[tw] OR service*[tw] OR therap*[tw] OR treatment*[tw] OR counsel*[tw] OR appointment[tw] OR prescri*[tw] OR service*[tw] OR platform*[tw] OR portal*[tw])) OR "Internet-Based Intervention"[MeSH] OR "Patient Portals"[Mesh] |
| AND |
| Support*[tw] OR encourag*[tw] OR incentiv*[tw] OR “increase use”[tw] OR teach*[tw] OR train*[tw] OR help*[tw] OR assist*[tw] OR engag*[tw] OR facilitat*[tw] OR promot*[tw] OR expedit*[tw] |
| AND |
| "primary care" [tw] OR "community-based provider*"[tw] OR "general practitioner*"[tw] OR GP[tiab] OR "family doctor"[tw] OR "Physicians, Primary Care"[MeSH] OR "General Practice"[MeSH] OR "General Practitioners"[MeSH] OR "Family Practice"[MeSH] OR "Primary Health Care"[MeSH] OR "ambulatory care"[MeSH] |
| AND |
| "national health service"[tiab] OR “national health service”[affiliation] OR nhs[tiab] OR nhs[Affiliation] OR Britain[tiab] OR Britain[Affiliation] OR British[tiab] OR GB[tiab] OR G.B.[tiab] OR GB[affiliation] OR G.B.[affiliation] OR “united kingdom”[tiab] OR “united kingdom”[affiliation] OR UK[tiab] OR U.K.[tiab] OR UK[affiliation] OR U.K.[affiliation] OR England[tiab] OR England[affiliation] OR London[Affiliation] OR London[tiab] OR “northern Ireland”[tiab] OR “northern ireland”[affiliation] OR “northern irish*”[tiab] OR Scotland[tiab] OR Scotland[affiliation] OR Scottish*[tiab] OR wales[tiab] OR wales[affiliation] OR welsh[tiab] OR United Kingdom[MeSH] OR Australia[MeSH] OR Australia[Affiliation] OR Australia*[tiab] OR Austria[MeSH] OR Austria*[tiab] OR Austria[affiliation] OR Belgium[MeSH] OR Belgium[affiliation] OR Belgium[tiab] OR Belgian[tiab] OR Czech Republic[MeSH] OR Czech[tiab] OR Czech[Affiliation] OR Denmark[MeSH] OR Denmark[tiab] OR Denmark[affiliation] OR Dane[tiab] OR Danish[tiab] OR Estonia[MeSH] OR Estonia[Affiliation] OR Estonia*[tiab] OR Finland[MeSH] OR Finland[Affiliation] OR Finland[tiab] OR Finnish[tiab] OR France[MeSH] OR France[Affiliation] OR France[tiab] OR French[tiab] OR Germany[MeSH] OR Germany[tiab] OR Germany[Affiliation] OR German*[tiab] OR Greece[MeSH] OR Greece[Affiliation] OR Greece[tiab] OR Greek[tiab] OR Hungary[MeSH] OR Hungary[tiab] OR Hungary[affiliation] OR Hungarian[tiab] OR Iceland[MeSH] OR Iceland[Affiliation] OR Iceland[tiab] OR Ireland[MeSH] OR Ireland[Affiliation] OR Ireland[tiab] OR Italy[MeSH] OR Italy[Affiliation] OR Italy[tiab] OR Italian[tiab] OR Latvia[MeSH] OR Latvia[Affiliation] OR Latvia*[tiab] OR Luxembourg[MeSH] OR Luxembourg[Affiliation] OR Luxembourg[tiab] OR Netherlands[MeSH] OR Netherlands[Affiliation] OR Netherlands[tiab] OR Dutch[tiab] OR Norway[MeSH] OR Norway[Affiliation] OR Norway[tiab] OR Norwegian[tiab] OR Poland[MeSH] OR Poland[Affiliation] OR Poland[tiab] OR Polish[tiab] OR Portugal[MeSH] OR Portugal[Affiliation] OR Portugal[tiab] OR Portuguese[tiab] OR Slovakia[MeSH] OR Slovakia[Affiliation] OR Slovak[tiab] OR “Slovak Republic”[Affiliation] OR Slovenia[MeSH] OR Slovenia[Affiliation] OR Slovenia*[tiab] OR Spain[MeSH] OR Spain[Affiliation] OR Spain*[tiab] OR Sweden[MeSH] OR Sweden[Affiliation] OR Sweden[tiab] OR Swedish[tiab] OR Switzerland[MeSH] OR Switzerland[Affiliation] OR Switzerland[tiab] OR Swiss[tiab] OR Canada[MeSH] OR Canada[Affiliation] OR Canada[tiab] OR Canadian[tiab] OR Chile[MeSH] OR Chile[Affiliation] OR Chile[tiab] OR Chilean[tiab] OR Colombia[MeSH] OR Columbia[Affiliation] OR Columbia*[tiab] OR Israel[MeSH] OR Israel[Affiliation] OR Israel*[tiab] OR Japan[MeSH] OR Japan[Affiliation] OR Japan*[tiab] OR Korea[MeSH] OR Korea[Affiliation] OR Korea*[tiab] OR Mexico[MeSH] OR Mexico[affiliation] OR Mexico[tiab] OR Mexican*[tiab] OR New Zealand[MeSH] OR New Zealand[Affiliation] OR New Zealand*[tiab] OR Turkey[MeSH] OR Turkey[Affiliation] OR Turkey[tiab] OR Turkish[tiab] OR United States[MeSH] OR United States[Affiliation] OR USA[Affiliation] OR US[Affiliation] OR New York[tiab] OR New York[Affiliation] OR Paris[affiliation] OR Paris[tiab] OR Dublin[affiliation] OR Dublin[tiab] OR Rome[affiliation] OR Rome[tiab] OR Berlin[affiliation] OR Berlin[tiab] OR united states[tiab] |
| **Results: 7682 – 4 internal duplicates = 7678** |

## Embase

| **Limits: English; 2010-present Search run: 18 June 2020** |
| --- |
| ((online:ti,ab,kw OR on-line:ti,ab,kw OR web:ti,ab,kw OR website:ti,ab,kw OR internet:ti,ab,kw OR network:ti,ab,kw OR digital:ti,ab,kw OR smartphone*:ti,ab,kw or "smart phone*":ti,ab,kw OR app:ti,ab,kw OR computer*:ti,ab,kw OR "mobile phone*":ti,ab,kw) AND (consult*:ti,ab,kw OR service*:ti,ab,kw OR therap*:ti,ab,kw OR treatment*:ti,ab,kw OR counsel*:ti,ab,kw OR appointment:ti,ab,kw OR prescri*:ti,ab,kw OR service*:ti,ab,kw OR platform*:ti,ab,kw OR portal*:ti,ab,kw)) OR 'web-based intervention'/exp |
| AND |
| Support*:ti,ab,kw OR encourag*:ti,ab,kw OR incentiv*:ti,ab,kw OR ”increase use”:ti,ab,kw OR teach*:ti,ab,kw OR train*:ti,ab,kw OR help*:ti,ab,kw OR assist*:ti,ab,kw OR engag*:ti,ab,kw OR facilitat*:ti,ab,kw OR promot*:ti,ab,kw OR expedit*:ti,ab,kw |
| AND |
| "primary care":ti,ab,kw OR "community-based provider*":ti,ab,kw OR "general practitioner*":ti,ab,kw OR GP:ti,ab,kw OR "family doctor":ti,ab,kw OR 'general practitioner'/exp OR 'general practice'/exp OR 'primary health care'/exp OR 'ambulatory care'/exp |
| AND |
| "national health service":ti,ab OR “national health service”:ff OR nhs:ti,ab OR nhs:ff OR Britain:ti,ab OR Britain:ff OR British:ti,ab OR GB:ti,ab OR G.B.:ti,ab OR GB:ff OR G.B.:ff OR “united kingdom”:ti,ab OR “united kingdom”:ff OR UK:ti,ab OR U.K.:ti,ab OR UK:ff OR U.K.:ff OR England:ti,ab OR England:ff OR London:ff OR London:ti,ab OR “northern Ireland”:ti,ab OR “northern ireland”:ff OR “northern irish*”:ti,ab OR Scotland:ti,ab OR Scotland:ff OR Scottish*:ti,ab OR wales:ti,ab OR wales:ff OR welsh:ti,ab OR 'united kingdom'/exp OR 'Australia'/exp OR Australia:ff OR Australia*:ti,ab OR 'austria'/exp OR Austria*:ti,ab OR Austria:ff OR 'Belgium'/exp OR Belgium:ff OR Belgium:ti,ab OR Belgian:ti,ab OR 'Czech Republic'/exp OR Czech:ti,ab OR Czech:ff OR 'Denmark'/exp OR Denmark:ti,ab OR Denmark:ff OR Dane:ti,ab OR Danish:ti,ab OR 'Estonia'/exp OR Estonia:ff OR Estonia*:ti,ab OR 'Finland'/exp OR Finland:ff OR Finland:ti,ab OR Finnish:ti,ab OR 'France'/exp OR France:ff OR France:ti,ab OR French:ti,ab OR 'Germany'/exp OR Germany:ti,ab OR Germany:ff OR German*:ti,ab OR 'Greece'/exp OR Greece:ff OR Greece:ti,ab OR Greek:ti,ab OR 'Hungary'/exp OR Hungary:ti,ab OR Hungary:ff OR Hungarian:ti,ab OR 'Iceland'/exp OR Iceland:ff OR Iceland:ti,ab OR 'Ireland'/exp OR Ireland:ff OR Ireland:ti,ab OR 'Italy'/exp OR Italy:ff OR Italy:ti,ab OR Italian:ti,ab OR 'Latvia'/exp OR Latvia:ff OR Latvia*:ti,ab OR 'Luxembourg'/exp OR Luxembourg:ff OR Luxembourg:ti,ab OR 'Netherlands'/exp OR Netherlands:ff OR Netherlands:ti,ab OR Dutch:ti,ab OR 'Norway'/exp OR Norway:ff OR Norway:ti,ab OR Norwegian:ti,ab OR 'Poland'/exp OR Poland:ff OR Poland:ti,ab OR Polish:ti,ab OR 'Portugal'/exp OR Portugal:ff OR Portugal:ti,ab OR Portuguese:ti,ab OR 'Slovakia'/exp OR Slovakia:ff OR Slovak:ti,ab OR "Slovak Republic":ff OR 'Slovenia'/exp OR Slovenia:ff OR Slovenia*:ti,ab OR 'Spain'/exp OR Spain:ff OR Spain*:ti,ab OR 'Sweden'/exp OR Sweden:ff OR Sweden:ti,ab OR Swedish:ti,ab OR 'Switzerland'/exp OR Switzerland:ff OR Switzerland:ti,ab OR Swiss:ti,ab OR 'Canada'/exp OR Canada:ff OR Canada:ti,ab OR Canadian:ti,ab OR 'Chile'/exp OR Chile:ff OR Chile:ti,ab OR Chilean:ti,ab OR 'Colombia'/exp OR Columbia:ff OR Columbia*:ti,ab OR 'Israel'/exp OR Israel:ff OR Israel*:ti,ab OR 'Japan'/exp OR Japan:ff OR Japan*:ti,ab OR 'Korea'/exp OR Korea:ff OR Korea*:ti,ab OR 'Mexico'/exp OR Mexico:ff OR Mexico:ti,ab OR Mexican*:ti,ab OR 'New Zealand'/exp OR "New Zealand":ff OR "New Zealand*":ti,ab OR 'Turkey'/exp OR Turkey:ff OR Turkey:ti,ab OR Turkish:ti,ab OR 'United States'/exp OR "United States":ff OR USA:ff OR US:ff OR "New York":ti,ab OR "New York":ff OR Paris:ff OR Paris:ti,ab OR Dublin:ff OR Dublin:ti,ab OR Rome:ff OR Rome:ti,ab OR Berlin:ff OR Berlin:ti,ab OR "United States":ti,ab |
| **Results: 5635 – duplicates = 2983** |

## CINAHL

| **Limits: English; 2010-present; Academic Journals Search run: 18 June 2020** |
| --- |
| (TI(online OR on-line OR web OR website OR internet OR network OR digital OR smartphone* or "smart phone*" OR app OR computer* OR "mobile phone*") AND TI(consult* OR service* OR therap* OR treatment* OR counsel* OR appointment OR prescri* OR service* OR platform* OR portal*)) OR (TI(online OR on-line OR web OR website OR internet OR network OR digital OR smartphone* or "smart phone*" OR app OR computer* OR "mobile phone*") AND AB(consult* OR service* OR therap* OR treatment* OR counsel* OR appointment OR prescri* OR service* OR platform* OR portal*)) OR (AB(online OR on-line OR web OR website OR internet OR network OR digital OR smartphone* or "smart phone*" OR app OR computer* OR "mobile phone*") AND AB(consult* OR service* OR therap* OR treatment* OR counsel* OR appointment OR prescri* OR service* OR platform* OR portal*)) OR  (AB(online OR on-line OR web OR website OR internet OR network OR digital OR smartphone* or "smart phone*" OR app OR computer* OR "mobile phone*") AND TI(consult* OR service* OR therap* OR treatment* OR counsel* OR appointment OR prescri* OR service* OR platform* OR portal*)) OR (MH "Patient Portals") |
| AND |
| TI(Support* OR encourag* OR incentiv* OR “increase use” OR teach* OR train* OR help* OR assist* OR engag* OR facilitat* OR promot* OR expedit*) OR AB(Support* OR encourag* OR incentiv* OR "increase use" OR teach* OR train* OR help* OR assist* OR engag* OR facilitat* OR promot* OR expedit*) |
| AND |
| (TI("primary care" OR "community-based provider*" OR "general practitioner*" OR GP OR "family doctor") OR AB("primary care" OR "community-based provider*" OR "general practitioner*" OR GP OR "family doctor")) OR (MH "Physicians, Family") OR (MH "Primary Health Care") OR (MH "Family Practice") OR (MH "Ambulatory Care") |
| AND |
| TI(“national health service”) OR AB(“national health service”) OR AF(“national health service”) OR TI(nhs) OR AB(nhs) OR AF(nhs) OR TI(Britain) OR AB(Britain) OR AF(Britain) OR TI(British) OR AB(British) OR TI(U.K.) OR AB(U.K.) OR AF(U.K.) OR TI(UK) OR AB(UK) OR AF(UK) OR TI(England) OR AB(England) OR AF(England) OR AF(London) OR TI(London) OR AB(London) OR TI(“Northern Ireland”) OR AB(“Northern Ireland”) OR TI(“Northern Irish*”) OR AB(“Northern Irish*”) OR TI(Scotland) OR AB(Scotland) OR AF(Scotland) OR TI(Scottish*) OR AB(Scottish) OR TI(Wales) OR AB(Wales) OR AF(Wales) OR TI(Welsh) OR AB(Welsh) OR (MH "United Kingdom+") OR (MH “Australia+”) OR AF(Australia) OR TI(Australia) OR AB(Australia) OR (MH “Austria”) OR TI(Austria) OR AB(Austria) OR AF(Austria) OR (MH “Belgium+”) OR AF(Belgium) OR TI(Belgium) OR AB(Belgium) OR TI(Belgian) OR AB(Belgian) OR (MH “Czech Republic+”) OR TI(Czech) OR AB(Czech) OR AF(Czech) OR (MH “Denmark”) OR TI(Denmark) OR AB(Denmark) OR AF(Denmark) OR TI(Dane) OR AB(Dane) OR TI(Danish) OR AB(Danish) OR (MH “Estonia+”) OR TI(Estonia) OR AB(Estonia) OR AF(Estonia) OR (MH “Finland+”) OR TI(Finland) OR AB(Finland) OR AF(Finland) OR TI(Finnish) OR AB(Finnish) OR (MH “France+”) OR TI(France) OR AB(France) OR AF(France) OR TI(French) OR AB(French) OR (MH “Germany+”) OR TI(German*) OR AB(German*) OR AF(Germany) OR (MH “Greece+”) OR TI(Greece) OR AB(Greece) OR AF(Greece) OR TI(Greek) OR AB(Greek) OR (MH “Hungary+”) OR AF(Hungary) OR TI(Hungary) OR AB(Hungary) OR TI(Hungarian) OR AB(Hungarian) OR (MH “Iceland+”) OR TI(Iceland) OR AB(Iceland) OR AF(Iceland) OR (MH “Ireland+”) OR TI(Ireland) OR AB(Ireland) OR AF(Ireland) OR (MH “Italy+”) OR TI(Italy) OR AB(Italy) OR AF(Italy) OR TI(Italian) OR AB(Italian) OR (MH “Latvia+”) OR TI(Latvia) OR AB(Latvia) OR AF(Latvia) OR (MH “Luxembourg+”) OR AF(Luxembourg) OR TI(Luxembourg) OR AB(Luxembourg) OR (MH “Netherlands+”) OR TI(Netherlands) OR AB(Netherlands) OR AF(Netherlands) OR TI(Dutch) OR AB(Dutch) OR (MH “Norway+”) OR TI(Norway) OR AB(Norway) OR AF(Norway) OR TI(Norwegian) OR AB(Norwegian) OR (MH “Poland+”) OR TI(Poland) OR AB(Poland) OR TI(Polish) OR AB(Polish) OR (MH “Portugal+”) OR TI(Portugal) OR AB(Portugal) OR AF(Portugal) OR TI(Portuguese) OR AB(Portuguese) OR (MH “Slovakia+”) OR TI(Slovakia) OR AB(Slovakia) OR AF(Slovakia) OR TI(“Slovak Republic”) OR AB(“Slovak republic”) OR AF(“Slovak Republic”) OR (MH “Slovenia+”) OR TI(Slovenia) OR AB(Slovenia) OR AF(Slovenia) OR (MH “Spain+”) OR TI(Spain*) OR AB(Spain*) OR AF(Spain) OR (MH “Sweden+”) OR TI(Sweden) OR AB(Sweden) OR AF(Sweden) OR TI(Swedish) OR AB(Swedish) OR (MH “Switzerland+”) OR TI(Switzerland) OR AB(Switzerland) OR AF(Switzerland) OR TI(Swiss) OR AB(Swiss) OR (MH “Canada+”) OR TI(Canada) OR AB(Canada) OR AF(Canada) OR TI(Canadian) OR AB(Canadian) OR (MH “Chile+”) OR TI(Chile) OR AB(Chile) OR AF(Chile) OR TI(Chilean) OR AB(Chilean) OR (MH “Colombia+”) OR AF(Colombia) OR TI(Colombia*) OR AB(Colombia*) OR (MH “Israel+”) OR TI(Israel*) OR AB(Israel*) OR AF(Israel) OR (MH “Japan+”) OR TI(Japan*) OR AB(Japan*) OR (MH “Korea+”) OR TI(Korea*) OR AB(Korea*) OR (MH “Mexico+”) OR TI(Mexico) OR AB(Mexico) OR AF(Mexico) OR TI(Mexican*) OR AB(Mexican*) OR (MH “New Zealand”) OR TI(“New Zealand”) OR AB(“New Zealand”) OR AF(“New Zealand”) OR (MH “Turkey+”) OR TI(Turkey) OR AB(Turkey) OR AF(Turkey) OR TI(Turkish) OR AB(Turkish) OR (MH "United States+") OR TI(“United States”) OR AB(“United States”) OR AF(“United States”) OR AF(USA) OR AF(US) OR TI(“New York) OR AB(“New York) OR AF(“New York) OR AF(Paris) OR TI(Paris) OR AB(Paris) OR TI(Dublin) OR AB(Dublin) OR AF(Dublin) OR TI(Rome) OR AB(Rome) OR AF(Rome) OR AF(Berlin) OR AB(Berlin) OR TI(Berlin) OR TI(“united states”) OR AB(“united states”) |
| **Results: 2899 – duplicates = 949** |

## Web of Science

| **Limits: English, 2010-present; Article, Review, early access** Refined by: COUNTRIES/REGIONS: **(will choose the countries on list) Search run: 18 June 2020** |
| --- |
| (TS=(online OR on-line OR web OR website OR internet OR network OR digital OR smartphone* or "smart phone*" OR app OR computer* OR "mobile phone*") AND TS=(consult* OR service* OR therap* OR treatment* OR counsel* OR appointment OR prescri* OR service* OR platform* OR portal*)) OR (TS=("Internet-Based Intervention*") OR TS=("Patient Portal*")) |
| AND |
| TS=(Support* OR encourag* OR incentiv* OR “increase use” OR teach* OR train* OR help* OR assist* OR engag* OR facilitat* OR promot* OR expedit*) |
| AND |
| TS=("primary care" OR "community-based provider*" OR "general practitioner*" OR "family doctor" OR "General Practice" OR "Family Practice” OR "Primary Health Care" OR "ambulatory care") |
| **Results: 5147 – duplicates = 1966** |

## Cochrane via Wiley (Issue 6 of 12, June 2020)

| **Limits: English, Added to Cochrane 2010-present – then published from 2010-2020; Search run: 18 June 2020** |
| --- |
| ((online OR on-line OR web OR website OR internet OR network OR digital OR smartphone* or "smart phone*" OR app OR computer* OR "mobile phone*"):ti,ab,kw AND (consult* OR service* OR therap* OR treatment* OR counsel* OR appointment OR prescri* OR service* OR platform* OR portal*):ti,ab,kw) OR [mh "internet-based intervention"] OR [mh "patient portals"] |
| AND |
| (Support* OR encourag* OR incentiv* OR “increase use” OR teach* OR train* OR help* OR assist* OR engag* OR facilitat* OR promot* OR expedit*):ti,ab,kw |
| AND |
| ("primary care" OR "community-based provider*" OR "general practitioner*" OR GP OR "family doctor"):ti,ab,kw OR [mh "Physicians, Primary Care"] OR [mh "General Practice"] OR [mh "General Practitioners"] OR [mh "Family Practice"] OR [mh "Primary Health Care"] OR [mh "ambulatory care"] |
| AND |
| "national health service":ti,ab OR nhs:ti,ab OR Britain:ti,ab OR British:ti,ab OR GB:ti,ab OR G.B.:ti,ab OR "united kingdom":ti,ab OR UK:ti,ab OR U.K.:ti,ab OR England:ti,ab OR London:ti,ab OR "northern Ireland":ti,ab OR "northern irish*":ti,ab OR Scotland:ti,ab OR Scottish*:ti,ab OR wales:ti,ab OR welsh:ti,ab OR [mh "United Kingdom" ] OR [mh Australia] OR Australia*:ti,ab OR [mh Austria] OR Austria*:ti,ab OR [mh Belgium] OR Belgium:ti,ab OR Belgian:ti,ab OR [mh "Czech Republic"] OR Czech:ti,ab OR [mh Denmark] OR Denmark:ti,ab OR Dane:ti,ab OR Danish:ti,ab OR [mh Estonia] OR Estonia*:ti,ab OR [mh Finland] OR Finland:ti,ab OR Finnish:ti,ab OR [mh France] OR France:ti,ab OR French:ti,ab OR [mh Germany] OR German*:ti,ab OR [mh Greece] OR Greece:ti,ab OR Greek:ti,ab OR [mh Hungary] OR Hungary:ti,ab OR Hungarian:ti,ab OR [mh Iceland] OR Iceland:ti,ab OR [mh Ireland] OR Ireland:ti,ab OR [mh Italy] OR Italy:ti,ab OR Italian:ti,ab OR [mh Latvia] OR Latvia*:ti,ab OR [mh Luxembourg] OR Luxembourg:ti,ab OR [mh Netherlands] OR Netherlands:ti,ab OR Dutch:ti,ab OR [mh Norway] OR Norway:ti,ab OR Norwegian:ti,ab OR [mh Poland] OR Poland:ti,ab OR Polish:ti,ab OR [mh Portugal] OR Portugal:ti,ab OR Portuguese:ti,ab OR [mh Slovakia] OR Slovak:ti,ab OR [mh Slovenia] OR Slovenia*:ti,ab OR [mh Spain] OR Spain*:ti,ab OR [mh Sweden] OR Sweden:ti,ab OR Swedish:ti,ab OR [mh Switzerland] OR Switzerland:ti,ab OR Swiss:ti,ab OR [mh Canada] OR Canada:ti,ab OR Canadian:ti,ab OR [mh Chile] OR Chile:ti,ab OR Chilean:ti,ab OR [mh Colombia] OR Columbia*:ti,ab OR [mh Israel] OR Israel*:ti,ab OR [mh Japan] OR Japan*:ti,ab OR [mh Korea] OR Korea*:ti,ab OR [mh Mexico] OR Mexico:ti,ab OR Mexican*:ti,ab OR [mh "New Zealand"] New Zealand*:ti,ab OR [mh Turkey] OR Turkey:ti,ab OR Turkish:ti,ab OR [mh "United States"] OR "united states":ti,ab OR "New York":ti,ab OR Paris:ti,ab OR Dublin:ti,ab OR Rome:ti,ab OR Berlin:ti,ab |
| **Results: 1208** (33 Reviews/1175 Trials) **– duplicates = 456** (13 Reviews/443 Trials) (of the trials: 212 CT.gov and 164 ICTRP) |

# Search of grey literature on digital facilitation in healthcare, all sectors

## Targeted searches of websites

**Table 10 Table of search terms and results for targeted searches of websites**

| **Website** | **Search number** | **Search terms** | **Search results** | **Removal of duplicates** |
| --- | --- | --- | --- | --- |
| King's fund | 1 | Online services | 35 |  |
|  | 2 | Digital online services | 12 | -2 |
|  | 3 | Digital facilitation | 10 |  |
|  | 4 | Online AND patients | 33 | -1 |
|  | 5 | Digital AND patients | 44 | -2 |
|  | 6 | Online access | 26 | -1 |
|  | 7 | Technology access | 54 | -4 |
| Royal College of GPs | 1 | Online services | 24 |  |
|  | 2 | Online AND patients | 3 |  |
|  | 3 | Digital access | 3 |  |
| Nuffield Trust | 1 | Online digital technology (filtered for Primary Care) | 8 |  |
|  | 2 | Online digital technology (no filter) | 41 |  |
| Health Foundation | 1 | Online | 4 |  |
|  | 2 | Digital | 1 |  |
|  | 3 | Technology | 10 |  |
| **Totals** | | | **308** | **-10** |
| **Total after removing duplicates** | | | **298** |  |

All searches limited to English, 2015-present

## Search of Health Management Information Consortium (HMIC) database

We searched the Health Management Information Consortium (HMIC) database using the following terms:

| **Limits: English, 2015-present** |
| --- |
| ((online OR digital OR virtual OR technolog*3) AND (uptake OR encourage OR "increase use" OR adopt*3 OR facilitat*3)).ti,ab [DT 2015-2020] |
| **Results = 27** |

## Search of academic literature on digital facilitation in non-healthcare sectors

We searched the literature on non-healthcare sectors via Google Scholar using the following search terms:

| **Limits: English, 2015-present** | | |
| --- | --- | --- |
| Sector | Search Terms | Search results |
| Tourism/Travel | (digital OR online OR smartphone OR “smart phone” OR application OR app) AND (airline OR flight OR travel OR accommodation OR tourism OR hotel) AND (uptake OR “increase use” OR facilitate) | 881,000 > Reviewed first 100 (sorted by relevance) for eligibility of which 2 were included for full-text extraction |
| Banking | (digital OR online OR smartphone OR “smart phone” OR application OR app) AND (banking OR banks OR finance) AND (uptake OR “increase use” OR facilitate) | 719,000 > Reviewed first 100 (sorted by relevance) ) for eligibility of which 3 were included for full-text extraction |
