# Supplementary material for: Digital Facilitation to Support Patient Access to Web-Based Primary Care Services: Scoping Literature Review
Source: J Med Internet Res. 2022 Jul 14;24(7):e33911. doi: 10.2196/33911 (PMC9335178; doi:10.2196/33911)
Supplement: Multimedia Appendix 2 [file jmir_v24i7e33911_app2.docx]

**Supplement B. Data fields in data extraction chart**

| **Field** | **Instructions to reviewer** |
| --- | --- |
| Study # |  |
| Full citation |  |
| Reviewer Initials |  |
| Article Type |  |
| Geographic focus of article |  |
| Article Topic | Briefly describe the main topic of the article. |
| Research question(s) (if applicable) |  |
| Research method(s) used (if applicable) |  |
| Study population | e.g. #practices, patients, etc. |
| Disease area/focus |  |
| What online service is the article about? | Brief description |
| What is the digital facilitation approach/activity? | Please describe what the digital facilitation approach/activity is. Please include a description of its size, scope, length of time in use/stage of development, if mentioned in the article. |
| Has the facilitation approach been implemented? | Routine use; Experimental use; Not implemented |
| Why was the digital facilitation effort undertaken? | Please describe why the facilitation effort was undertaken (e.g. any drivers). This could include factors related to Covid-19. |
| How does the digital facilitation happen? | Please describe how the digital facilitation happens. |
| Who delivers the digital facilitation approach? | Please describe who is involved in the facilitation process. Please include any staff training activities as well as direct interactions with patients/members of the public. |
| Who is receiving the activity (population targeted)? | Please describe the target population. Mention any vulnerable groups that are targeted by the digital facilitation efforts, and describe how they were targeted. Examples of vulnerable groups include older adults, non-native/non/ English speakers, low-income populations, people with low literacy levels, BAME communities and rural populations |
| Vulnerable Group (Y/N) | (Examples of vulnerable groups include older adults, non-native/non/ English speakers, low-income populations, people with low literacy levels, BAME communities and rural populations.) |
| Barriers to digital facilitation | Please describe any barriers to the facilitation effort. This could include factors related to Covid-19. |
| Enablers for digital facilitation | Please describe anything that enabled or helped the digital facilitation effort. This could include factors related to Covid-19. |
| Evidence of effectiveness | Was any form of evaluation conducted? Please describe any evidence cited in the article to support the efficacy (or lack of efficacy) of the facilitation approach. |
| Evidence of cost/resource use | What costs/resources were used to deliver the facilitation programme? |
| Evidence of cost-effectiveness | Was a cost-effectiveness evaluation conducted? Please describe any evidence related to the cost-effectiveness of the approach. |
| Evidence related to Covid-19 | Did the article mention any other factors/responses to Covid-19 related to the digital facilitation effort that have not already been captured in the template? |
| Any evidence of harm from the facilitation effort? |  |
| Any other relevant information |  |
| *Quality of the research* | |
| Quality of evidence source | Has the digital facilitation effort been evaluated? If so, how? If the evidence presented in the article is not from an evaluation, please describe the source of the evidence, noting any comments on quality |
| Clarity of aims | Is the question(s) the paper seeks to answer clear and well described? |
| Clarity of methods | Are the method(s) the paper uses clear and well described? For example, if it's a survey, do they cite their sampling strategy and response rate? |
| Quality and comprehensiveness of work | How comprehensive is the publication? Does it draw on or at least seems to be informed by a robust evidence base? |
| Conflicts of interest | Are there any potential conflicts of interest amongst authors that could have influenced findings? |
| Other comments on quality: Please note any other reflections on the article's quality. |  |
| Notes for additional searches | Please note any additional terms or comments that you feel might be useful for snowballing or grey literature searches |
