# Supplementary material for: Digital Facilitation to Support Patient Access to Web-Based Primary Care Services: Scoping Literature Review
Source: J Med Internet Res. 2022 Jul 14;24(7):e33911. doi: 10.2196/33911 (PMC9335178; doi:10.2196/33911)
Supplement: Multimedia Appendix 3 [file jmir_v24i7e33911_app3.docx]

Supplement C presents a full list of publications included in the scoping review of the literature. We defined a typology of online services supported by digital facilitation. These include: (1) websites (e.g. to access online health records or health information); (2) patient portals and personalized practice tools (e.g. to order repeat prescriptions or book appointments online); (3) health apps (e.g. for self-management of health conditions, or to access online health information or patient portals); and (4) screening programs and customized health information on tablets.

We also developed a typology of digital facilitation approaches. The approaches are most frequently aimed at patients but may also target primary care staff. The approaches include:

1. Promotion: a broad category of digital facilitation that captures ways of raising awareness of and knowledge about digital services, endorsements of specific digital services to patients, and methods of encouraging patients to use them;
2. Training and education: education or training to help patients acquire technical skills to use digital services or to help patients understand what features of a digital service can be most helpful to them.
3. Guidance and support: Ongoing help in using digital services provided by clinicians or other primary care staff to patients.

Supplement C. Full list of publications included in scoping review of the literature.

| Digital facilitation type | Reference | Purpose of study | Online service supported | Geographic focus | Sector | Study design | Study population | Disease area/focus |
| --- | --- | --- | --- | --- | --- | --- | --- | --- |
| Promotion | Aarts JW, et al. (2015)[36] | Explores experiences of professionals and patients with the implementation of an online infertility community into a clinic’s care practice. | Website | Netherlands | Secondary care | Qualitative study | N=1 practice site: Interviews with N=6 Healthcare professionals and N=7 patients | Infertility |
| Promotion | Abbott-Garner P, et al. (2019)[60] | This study aimed to assess the impact of 3 interventions on personal eHealth readiness | Patient portal | UK | Primary care | Prospective cohort study | N=1388 households; from 78 postcodes served by 78 different GP practices | N/A |
| Promotion | Ayre J, et al. (2020)[37] | To explore GP perceptions of how diabetes app features could help GPs better support their patients from culturally diverse backgrounds | Health app | Australia | Primary care | Qualitative study | N=25 GPs | Diabetes |
| Promotion | Baumeister H, et al. (2015) | To look at whether patients accept online intervention for pain, and impacts of an acceptance facilitating intervention on acceptance of internet-based psychological pain intervention | Website | Germany | Healthcare | RCT | N=104; Patients treated in pain centres | Pain |
| Promotion | Byambasuren O, et al. (2020)[65] | Mobile health (mHealth) apps and their usability as non-drug interventions in primary care | Health app | Australia | Primary care | Pre/post study | N=40, GPs | N/A |
| Promotion | Byczkowski TL, et al.et al. (2011)[116] | To understand the use of internet-based portal among families of children with chronic diseases | Patient portal | USA | Secondary care | Retrospective observational study | N=1960; Children with chronic diseases (1900 families) | Diabetes mellitus, juvenile idiopathic arthritis, cystic fibrosis |
| Promotion | Carter M, et al. (2018)[34] | To evaluate the feasibility, acceptability and effectiveness of webGP | Patient portal | UK | Primary care | Mixed-methods; Qualitative and Retrospective case series | N=6 practices; N=81 patients; N=10 interviews (5 GPs, 5 administrators); N=20 GPs completing case reports | N/A |
| Promotion | Castle-Clarke S (2018)[66] | Report looking at the potential impact of technology on health services | Health app | UK | Healthcare | Narrative review | N/A | N/A |
| Promotion | Delbanco T, et al. (2012)[80] | To evaluate the impact of facilitating patient access to visit notes over secure internet portal | Patient portal | USA | Primary care | Prospective cohort study | N=13,669; 105 primary care providers and 13,564 patients | N/A |
| Promotion | Greenhalgh T, et al. (2010)[33] | To evaluate policy, implementation and patient and carer experiences with internet accessible electronic health records | Patient portal | England | Healthcare | Mixed method case study | N=216 (for interviews); 56 patients and 160 policy makers, project managers and clinical staff | N/A |
| Promotion | Hassett L, et al. (2020)[73] | To examine the impact of prescribed digitally-enabled rehabilitation for people with mobility issues | Health app | Australia | Healthcare | RCT | N=300 patients | Mobility limitation |
| Promotion | Hoffmann M, (2019)[71] | Assessing how to integrate mental health online video consultations in primary care | Website | Germany | Primary care | Qualitative study | N=19 GPs | Mental health |
| Promotion | Irizarry T, et al. (Jun 2015)[21] | Examining how to improve patient engagement with portals | Patient portals | N/A | Primary care | Narrative review | N/A | N/A |
| Promotion | Lillevoll KR, et al. (2014)[93] | To look at the uptake and adherence of internet-based mental health intervention with tailored email reminders in senior high schools | Website | Norway | Healthcare | RCT | N=707; Students in senior high school | Mental health; Depressive symptoms |
| Promotion | Lin JX, et al. (2018)[82] | To understand patients' willingness to engage in internet- and mobile-based interventions for chronic pain and how uptake of interventions can be improved | Website; Health app | Germany | Primary care | RCT | N=115; Patients with chronic pain | Chronic pain |
| Promotion | Lopez Segui F, et al. (2018)[61] | Pilot study on implementation of website that allows primary care providers to prescribe app in field of health and social services | Health app | Spain | Primary care | Mixed methods; Qualitative, Prospective case study | N=47 (Questionnaire); 17 doctors and 30 patients | Asthma; Chronic illness; Alcohol consumption |
| Promotion | Mafi JN, (2016)[117] | To investigate how to get patients to view clinician notes on a patient portal | Patient portal | USA | Primary care | Prospective cohort study | N=15,360 patients | N/A |
| Promotion | Mahoney N, et al. (2017)[63] | To understand the internal factors that affect the ability of healthcare organizations to implement an internet-based intervention | Website | USA | Primary care | RCT | N=369; Adolescents | Depression |
| Promotion | Mares ML, et al. (2016)[38] | To examine the perspective of clinicians that implement a digital intervention | Health app | USA | Primary care | Mixed methods; Prospective case studies | N=53; Clinicians | Substance use disorder |
| Promotion | Creber RM, et al. (2019)[118] | How to engage hospitalized patients with personalized health information | Patient portal | USA | Secondary care | RCT | N=426 patients | N/A |
| Promotion | Nguyen AD, et al. (2019)[31] | To obtain GP perspective on patient use of mobile health apps and their own role in the use of health apps | Health app | Australia | Primary care | Qualitative study | N=10; GPs | Chronic conditions |
| Promotion | Patel V, et al. (2015)[85] | To understand patients' use of tablets during clinical visit, time spent in waiting room and use of health information resources | Screening programs and customized health information on tablets | USA | Primary care | Cross-sectional | N=89; 84 patients and 5 healthcare providers | N/A |
| Promotion | Plaete J, et al. (2015)[35] | To explore the feasibility of an eHealth program | Screening programs and customized health information on tablets | Belgium | Primary care | Qualitative study | N=62; GPs | Chronic conditions |
| Promotion | Radovic A, et al. (2019)[79] | Pre-implementation study looking at primary care providers perspectives on implementation of technology prescription for treatment of young peoples' mental health issues to address barriers to | Website | USA | Primary care | Qualitative study | N=14+; Primary care providers and adolescent patients with depression | Depression and anxiety |
| Promotion | Ricciardi L, (2013)[104] | Reviewing recent evidence in support of consumer e-health to increase patient engagement | Website | USA | Healthcare | Narrative review | N/A | N/A |
| Promotion | Ronda MCM, et al. (2018)[69] | To gain insight on opinions and working methods of providers after using diabetes web portal for 4 years | Patient portal | Netherlands | Primary care | Cross-sectional | N=128; Physicians and nurses from general practice and outpatient clinics | Diabetes |
| Promotion | Sprenger M, et al. (2017)[39] | To explore the intention of healthcare professionals to use and recommend e-mental health apps, how this intention is influenced, which group of healthcare professionals may be most accessible to recommend app and for which tasks healthcare professionals rate apps to be most useful | Health app | USA; Spain; Switzerland | Primary care | Cross-sectional | N=131; Healthcare professionals working in maternity care | Maternal mental health |
| Promotion (for patient); Training; Guidance and support (for providers) | Molleda L, et al. (2017)[64] | To understand feasibility and acceptability of implementing an internet-based intervention | Website | USA | Primary care | Qualitative study | N=48; Hispanic adolescents and their parents | Behavioural health (drug use, sexual health) |
| Promotion (for patients); Training (for provider) | Webb MJ, et al. (2018)[68] | To assess implementation of a health and lifestyle screening app for young people | Screening programs and customized health information on tablets | Australia | Primary care | Case study | N=99; 4 GPs, 10 support staff and 85 young people aged 14-25 | N/A |
| Promotion; Guidance and support | Lipschitz J, et al. (2019)[72] | To understand current use of mHealth for mental illness, along with factors that affect use | Health app | USA | Primary care | Cross-sectional study | N=149; Patients with mental health conditions in facility that treats veterans | Mental health; Unipolar depression; Anxiety disorders; PTSD |
| Promotion; Guidance and support | O'Cathain A, et al. (2016)[99] | To understand views on intervention where trained staff implement telehealth intervention | Website | UK | Primary care | Qualitative study | N=53; 21 primary care staff, 8 NHS Direct staff and 24 patients | Depression and cardiovascular risk |
| Promotion; Guidance and support | Reynolds J, et al. (2015)[74] | To develop a conceptual framework to support the use of e-mental health resources in primary care | Websites | Australia | Healthcare | Narrative literature review | N/A | Mental health |
| Promotion; Guidance and support; Training | Longacre M, et al. (2018)[76] | To look at themes from the introduction of an mHealth intervention for pediatric cystic fibrosis in a hospital in Sweden | Health app | Sweden | Secondary care | Retrospective qualitative case study | Unclear | Paediatric cystic fibrosis |
| Promotion; Guidance and support; Training | Miller KE, et al. (2019)[49] | To evaluate use and perceptions of apps among primary care mental health providers and identify challenges with implementation | Health app | USA | Primary care | Cross-sectional | N=220; Primary care mental health providers | Mental health |
| Promotion; Training | Coulter A and Mearns B (2016)[75] | Report on improving the quality and efficiency of healthcare. Discusses patient engagement through health information technologies | Patient portal; Website | UK | Healthcare | Narrative review | N/A | N/A |
| Promotion; Training | du Pon E, et al. (2020)[119] | To investigate whether a group education program can improve the use of an online care platform in patients | Website | Netherlands | Primary care | RCT | N=203 patients | Type-2 Diabetes |
| Promotion; Training | Ebert D, et al. 2015)[41] | To examine effects of a video training session on internet-based intervention uptake | Website | Germany | Primary care | RCT | N=128 patients | Depression |
| Promotion; Training | Slevin P, et al. (2020)[55] | To understand barriers and facilitators that providers perceive for the use of digital health technologies in those with COPD | Health app | Ireland | Primary care | Qualitative study | N=32; Providers (GPs and respiratory therapists) | COPD |
| Promotion; Training (for providers) | Raza Khan U, et al. (2019)[102] | To understand adoption of patient portal, including how to increase uptake of patient portals | Patient portal | Australia | Primary care | Case study | N=10; Primary care staff | N/A |
| Promotion; Training; Guidance and support | Castle-Clarke S and Imison C (2016)[70] | Review of evidence on digital technology and its impacts on primary care patients and the NHS | Health app; Website; Patient portal | UK | Healthcare | Case studies | N/A | N/A |
| Training | Abidi S, et al. (2018)[91] | Evaluation of a computerized decision support platform, with a patient-facing element | Website | USA | Primary Care | Qualitative study | N=21; 10 primary care providers, 11 patients | Diabetes |
| Training | Bernhard G, et al. (2018)[62] | To identify healthcare professional and patient preferences regarding web-based medication platform | Patient portal | Germany | Primary care | Qualitative study | N=48; 25 patients, 13 GPs, 10 health care assistants | Diabetes |
| Training | Chan B, et al. (2018)[120] | Examines the characteristics of patients who use online portals versus those that don’t | Patient portal | USA | Primary care | Cross-sectional study | N=17,699 patient records | N/A |
| Training | Collins B (2020)[121] | Case studies of 4 digital innovations | Screening program and customized health information on tablet | UK and Nordic countries | Healthcare | Case studies | N=4 Case study sites | N/A |
| Training | Hjelmager DM, (2019)[40] | Implementing online information material for patients with low back pain in general practice | Website | Denmark | Primary care | Qualitative study | N=8 GPs | Low back pain |
| Training | Lyles CR, et al. (2016)[53] | To understand specific barriers that African American and Latino patients face in accessing electronic health records and patient portals | Patient portal; Health app | USA | Healthcare | Qualitative study | N=87; English-speaking Latino and African American patients | N/A |
| Training | Lyles C R, et al. (2019)[81] | To test the effectiveness of delivering online, video-based training to use a patient portal | Patient portal | USA | Primary care | RCT | N=93; Patients with chronic disease | Chronic diseases |
| Training | Moult, A et al. (2018)[52] | To explore how older adults use the internet to self-manage distress | Website | UK | Primary care | Qualitative study | N=18**;** Adults 65 or older | Anxiety and depression; distress |
| Training | Rodgers M, et al. (2019)[92] | Rapid evidence synthesis to inform NHS England digital first primary care policy | Patient portal | UK | Primary care | Rapid evidence assessment | N/A | N/A |
| Training | Tieu L, et al. (2015)[54] | To understand patient and caregiver perspectives on online patient portal use | Patient portal | USA | Healthcare | Qualitative study | N=16; Patients in safety net hospital with chronic diseases and carers | Chronic diseases |
| Training (both for patient and provider); Promotion (for patient) | Ramsey A, et al. (2018)[42] | To determine the feasibility, acceptability and preliminary effectiveness of assigning designated staff to assess adolescents with patient portal sign-up, as well as investigating patient satisfaction and preferences about future communication around health issues | Patient portal | USA | Primary care | Cross-sectional | N=96; Patients 13-25 years old in urban clinic | N/A |
| Training (for patients); Guidance and support (for patients and providers) | Ford JH, et al. (2015)[83] | To look at challenges and problems associated with sustained use of mHealth app for addiction support | Health app | USA | Healthcare | Qualitative study | N=44; Clinicians and administrators providing drug and alcohol treatment | Addiction |
| Training (for providers and patients) | Sieck CJ, et al. (2017)[84] | To examine the experience of physicians and patients that are experienced in using ambulatory portals with regard to perceived benefits and challenges, and the use of secure messages through the portal | Patient portal | USA | Primary care | Qualitative study | N=42; 29 patients and 13 primary care providers | Cardiopulmonary conditions |
| Training; Guidance and support | Apter AJ, et al. (2019)[45] | Examined effects of training patients in use of patient portal | Patient Portal | USA | Primary Care | RCT | N=300 | Asthma |
| Training; Guidance and support | Talboom-Kamp E P, et al. (2017)[122] | To analyze factors that support use of web-based self-management platform for COPD, focusing on how levels of integration with primary care and assistance from research nurse influence use | Website | Netherlands | Primary care | Prospective cohort study | N=215; Patients with COPD | COPD |
| Training; Promotion | Grossman LV, et al. (2019)[32] | To systematically review the impact of interventions designed to: (1) increase patient portal use or predictors of use in vulnerable patient populations, or (2) reduce disparities in use. | Patient portals | N/A | Primary Care | Systematic literature review | N/A – systematic review | N/A |
| Training; Promotion | North F, et al. (2011)[50] | To provide quantitative evidence on impact of video promotion on patient portal adoption | Patient portal | USA | Primary care | RCT | N=12,050 (office appointments) | N/A |
| Training; Promotion | Phelps RG, et al. (2014)[43] | To look at registration for an online patient portal, and endurance of appeal for different patient groups | Patient portal | UK | Tertiary care | Longitudinal observational study | N=11,352; patients with chronic kidney disease | Chronic kidney disease |
| Guidance and support | Baumeister H, et al. (2014)[48] | To look at whether diabetes patients accept internet-based interventions for depression, and the impacts of an acceptance facilitating intervention | Website | Germany | Healthcare | RCT | N=141; Diabetes patients in inpatient and outpatient rehabilitation clinics | Diabetes; Depression |
| Guidance and support | Cafazzo JA, et al. (2012)[123] | Pilot study to design, develop and pilot use of mHealth intervention for adolescents with Type 1 diabetes. | Health app | Canada | Healthcare | Qualitative study | N=20; Adolescents with Type 1 diabetes and their parents (12 interviewed) | Adolescent Type 1 diabetes |
| Guidance and support | Careyva B, et al. (2016)[98] | How primary care directors engage patients in online services | Patient portals | USA | Primary care | Cross-sectional study | N=102, practice-based research network directors | N/A |
| Guidance and support | Lin J, et al. (2017)[87] | To compare guided and unguided online acceptance and commitment therapy | Website | Germany | Healthcare | RCT | N=302; | Chronic pain |
| Guidance and support | Littlewood E, et al. (2015)[90] | To assess new health technology around computerized therapy for depression | Website | UK | Primary care | Qualitative study | N=11; GPs | Depression |
| Guidance and support | Ma J, et al. (2013)[88] | To evaluate the acceptance and use of a self-monitoring website for weight loss, comparing a coach-led and self-guided version of program | Website | USA | Healthcare | Secondary analysis of data from RCTs | N=64; Overweight adults with pre-diabetes | Weight loss; Pre-diabetes |
| Guidance and support | Possemato K, et al. (2016)[89] | To evaluate the feasibility and effectiveness of an app for PTSD with clinician support and without clinician support, including use and engagement | Health app | USA | Primary care | RCT | N=20; Veterans with PTSD from military related trauma | PTSD |
| Guidance and support | Ray JM,et al. (2017)[124] | To understand veteran's preferences for a peer-supported engagement to help develop peer support protocols | Website | USA | Healthcare | Qualitative study | N=24; Veterans with anxiety and depression | Depression and anxiety |
| Guidance and support | Richardson CR, et al. (2010)[125] | Evaluating adherence in an internet-mediated walking program | Website | USA | Healthcare | RCT | N=324 patients | Obesity |
| Guidance and support | van Middelaar T, et al. (2018)[56] | To assess what factors influence initial and sustained engagement with an internet platform for self-management of cardiovascular disease risk in primary care | Website | Netherlands | Primary care | Qualitative study | N=20; Patients over 65 with risk of cardiovascular disease | Cardiovascular disease |
| Guidance and support | Zarski A, et al. (2018)[78] | To understand the motivational and volitional factors associated with adherence to an online intervention for depression prevention | Website | Germany | Healthcare | Secondary analysis of data from RCTs | N=101; Patients with sub-clinical depression | Sub-clinical depression |
| Guidance and support | Zarski A, et al. (2016)[94] | To look at the influence of different guidance formats on adherence to online support for stress management | Website | Germany | Healthcare | Secondary analysis of data from RCTs | N=395; Employed adults with stress | Stress |
| Guidance and support (for providers) | Hale K, et al. (2015)[126] | To present framework to identify trustworthy apps for patients | Health app | Australia | Healthcare | Narrative review | N/A | Type 2 diabetes and other chronic conditions |
| Guidance and support; Promotion | Cowie J, et al. (2018)[58] | Evaluation of eConsult, including impacts on GP surgery | Patient portal | UK | Primary care | Mixed-methods; Qualitative and Retrospective case series | N=11 GP practices and their patients | N/A |
| Guidance and support; Promotion | van Beurden SB, et al. (2018)[67] | To explore factors that influence primary care patients' initial uptake and continued use of weight management websites | Website | England | Primary care | Qualitative study | N=20; Patients that are overweight and want to lose weight | Overweight/Obesity |
| Guidance and support; Training | Nijland N, et al. (2011)[51] | Process analysis of use of web-app for diabetes self-care over 2 year period | Website | Netherlands | Primary care | Mixed methods; Qualitative, Prospective case study | N=50; Patients with diabetes enrolled to use app | Type 2 diabetes |
| N/A | Cameron, G, et al. (2019)[96] | Report on technology likely to shape health and social care in England | N/A | UK | Health – all sectors | Narrative review | N/A | N/A |
| N/A | Castle-Clarke S, et al. (2016)[127] | Briefing examining how technology can underpin changes in primary care | Patient portal; Website | UK | Healthcare | Qualitative study; Case studies | N/A | N/A |
| N/A | Oliveira Hashiguchi T (2020)[100] | Working paper on the use of telemedicnie in OECDC countries | Health app | OECD countries | Healthcare | Mixed method: Qualitative, Narrative review, Cross-sectional study | N/A | N/A |
| N/A | Greenhalgh T, et al. (2017)[97] | Framework for evaluating the success of a technology-supported health or social care program | Various | USA | Secondary care | Case study | N=6 case studies | N/A |
| N/A | Hardiker NR and Grant MJ (2010)[95] | Review public engagement with eHealth | Website; Patient portal; Health app | International | Healthcare | Rapid evidence assessment | N/A | N/A |
| N/A | Honeyman M, et al. (2016)[128] | Reviewing digitalization in healthcare | Various | UK | Healthcare | Narrative review | N/A | N/A |
| N/A | Hutchings R (2020)[115] | Briefing discussing the impact of Covid-19 on how digital technologies are used in the NHS | Health app; Patient portal | UK | Healthcare | Narrative review | N/A | N/A |
| N/A | Maguire D, et al. (2018)[129] | Report examining how to support health and social care organizations that are looking to undertake large-scale digital change | Various | UK | Healthcare | Case studies | N/A | N/A |
| N/A | Rosen R, (2019)[103] | Briefing that presents ideas on how general practice can be provided with current shortage of GPs | Patient portal; Health app | UK | Primary care | Qualitative study | N/A | N/A |
| N/A | Weppner WG, et al. (2010)[101] | To evaluate a web-based shared medical record for older patients with diabetes | Patient portal | USA | Healthcare | Retrospective cohort study | N=6185; Older adults with diabetes | Diabetes |
| N/A | Yamin CK, (2011)[130] | Examining the digital divide in adoption of personal health records | Patient portal | USA | Healthcare | Cross-sectional | N=75,056 patients | N/A |
